# Crystal structure of l-2-keto-3-deoxyfuconate 4-dehydrogenase reveals a unique binding mode as a α-furanosyl hemiketal of substrates

**DOI:** 10.1038/s41598-024-65627-8

**Published:** 2024-06-25

**Authors:** Miyu Akagashi, Seiya Watanabe, Sebastian Kwiatkowski, Jakub Drozak, Shin-ichi Terawaki, Yasunori Watanabe

**Affiliations:** 1 Department of Bioscience, Graduate School of Agriculture, Ehime University, Matsuyama, Ehime Japan; 2 Faculty of Agriculture, Ehime University, Matsuyama, Ehime Japan; 3 Center for Marine Environmental Studies (CMES), Ehime University, Matsuyama, Ehime Japan; 4 Department of Metabolic Regulation, Institute of Biochemistry, Faculty of Biology, University of Warsaw, Warsaw, Poland; 5 Division of Structure Analysis of Protein Complex, Proteo-Science Center (PROS), Ehime University, Matsuyama, Ehime Japan; 6 Faculty of Science, Yamagata University, 1-4-12 Kojirakawa-machi, Yamagata, Yamagata 990-8560 Japan

**Keywords:** 2-keto-3-deoxysugar acid, Dehydrogenase, Short-chain dehydrogenase/reductase, Substrate specificity, Furanosyl hemiketal, Molecular evolution, Biochemistry, Structural biology

## Abstract

l-2-Keto-3-deoxyfuconate 4-dehydrogenase (l-KDFDH) catalyzes the NAD^+^-dependent oxidization of l-2-keto-3-deoxyfuconate (l-KDF) to l-2,4-diketo-3-deoxyfuconate (l-2,4-DKDF) in the non-phosphorylating l-fucose pathway from bacteria, and its substrate was previously considered to be the acyclic α-keto form of l-KDF. On the other hand, BDH2, a mammalian homolog with l-KDFDH, functions as a dehydrogenase for *cis*-4-hydroxy-l-proline (C4LHyp) with the cyclic structure. We found that l-KDFDH and BDH2 utilize C4LHyp and l-KDF, respectively. Therefore, to elucidate unique substrate specificity at the atomic level, we herein investigated for the first time the crystal structures of l-KDFDH from *Herbaspirillum huttiense* in the ligand-free, l-KDF and l-2,4-DKDF, d-KDP (d-2-keto-3-deoxypentonate; additional substrate), or l-2,4-DKDF and NADH bound forms. In complexed structures, l-KDF, l-2,4-DKDF, and d-KDP commonly bound as a α-furanosyl hemiketal. Furthermore, l-KDFDH showed no activity for l-KDF and d-KDP analogs without the C5 hydroxyl group, which form only the acyclic α-keto form. The C1 carboxyl and α-anomeric C2 hydroxyl groups and O5 oxygen atom of the substrate (and product) were specifically recognized by Arg148, Arg192, and Arg214. The side chain of Trp252 was important for hydrophobically recognizing the C6 methyl group of l-KDF. This is the first example showing the physiological role of the hemiketal of 2-keto-3-deoxysugar acid.

## Introduction

**Figure 1:**
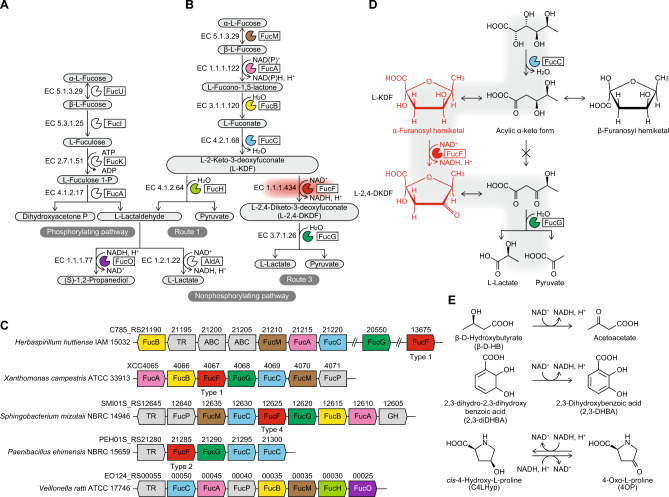
Physiological role of l-KDFDH. Phosphorylating (**A**) and nonphosphorylating (**B**) pathways of l-fucose metabolism from bacteria and the schematic gene cluster (**C**). Homologous genes are indicated in the same color and correspond to (**A**,**B**). (**D**) Schematic conversion of l-fuconate to pyruvate and l-lactate via l-KDF and l-2,4-DKDF intermediates by the consecutive actions of FucC, FucF, and FucG. The present study revealed that l-KDFDH, encoded by the FucF gene, utilizes l-KDF as α-furanosyl hemiketal, but not the acyclic α-keto form (previously proposed). (**E**) Schematic reactions by BDH2, a mammalian homolog of bacterial l-KDFDH.

l-Fucose is naturally present in the cell envelope of numerous prokaryotes and in glycoproteins of eukaryotes as a constituent of exopolysaccharides, and is the sole carbon and energy source for many microorganisms via two separate pathways. The majority of bacteria, including *Escherichia coli*, possess the well-known “phosphorylating” pathway in which l-fucose is converted into dihydroxyacetone phosphate and l-lactaldehyde by l-fucose mutarotase (EC 5.1.3.29; FucU), l-fucose isomerase (FucI), l-fuculokinase (FucK), and l-fuculose 1-phosphate aldolase (FucA) (Fig. 1A). l-Lactaldehyde is further converted to l-lactate or 1,2-propanediol by l-lactaldehyde dehydrogenase (AldA) and lactaldehyde:propanediol oxidoreductase (FucO) under aerobic and anaerobic conditions, respectively^1^. These metabolic genes often form a single transcriptional unit in bacterial genomes, such as the *fucIPKU*/*fucAR* operons, together with the (underlined) transporter and regulator.

Alternatively, there are so-called “nonphosphorylating” pathways of l-fucose metabolism from bacteria, in which l-fucose is commonly converted to l-2-keto-3-deoxyfuconate (l-KDF) intermediates by the consecutive actions of l-fucose mutarotase (FucM), l-fucose 1-dehydrogenase (FucA), l-fucono-1,5-lactonase (FucB), and l-fuconate dehydratase (FucC) (Fig. 1B). l-KDF is subsequently cleaved through an aldol-cleavage reaction to pyruvate and l-lactaldehyde by l-KDF aldolase (FucH) (route 1)^2^ or converted into pyruvate and l-lactate by the sequential actions of l-KDF 4-dehydrogenase (l-KDFDH) (EC 1.1.1.434; FucF) and l-2,4-diketo-3-deoxyfuconate (l-2,4-DKDF) hydrolase (FucG) (route 3)^3,4^; the route 2 pathway is specific for pentose including d-xylose, l-arabinose, and d-arabinose, and D/l-2-keto-3-deoxypentonate (KDP) intermediates are commonly converted to α-ketoglutarate by the consecutive actions of dehydratase (EC 4.2.1.43 and EC 4.2.1.141) and dehydrogenase (EC 1.2.1.26). These metabolic genes are also clustered in microorganism genomes (Fig. 1C). In the homologous route to the latter for l-rhamnose metabolism^5^, the l-2-keto-3-deoxyrhamnonate (l-KDR) intermediate at the branch point loses chirality at C4 by the dehydrogenase (LRA5), by which the same intermediate as l-2,4-DKDF (l-2,4-diketo-3-deoxyrhamnonate) is produced (Fig. S1). This metabolic fate is also hydrolyzed to pyruvate and l-lactate by the hydrolase (LRA6), which is phylogenetically close to FucG. Recently, non-phosphorylating l-fucose and/or l-rhamnose pathway(s) were shown to play important physiological roles in the gastrointestinal pathogen *Campylobacter jejuni*^6^ and (previously unculturable) marine bacteria of the SAR202 clade^7^.

**Figure 2:**
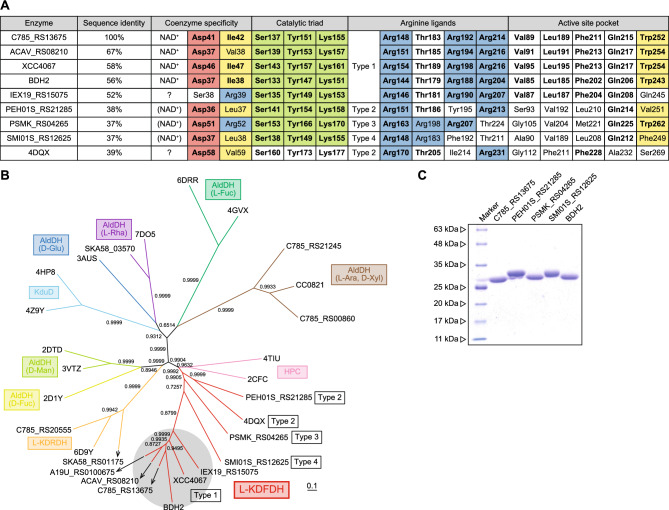
Phylogenetic analysis of l-KDFDH. (**A**) Comparative sequence analysis of l-KDFDH. Bold letters are identical to l-KDFDH from *H. huttiense*. Characteristic aspartate and hydrophobic residues and the arginine residue for NAD^+^- and NADP^+^-dependent enzymes are shadowed in red, yellow, and blue, respectively. Coenzyme specificity in parentheses was elucidated in the present study. A Ser-Tyr-Lys motif of the catalytic triad, completely conserved among SDR superfamily enzymes, is shadowed in green-yellow. A pattern of arginine ligands (shadowed in blue) that recognizes the C1 carboxyl group of l-KDF is classified into types 1 ~ 4. Among active site pockets, the highly conserved hydrophobic residue shadowed in yellow was close to the C6 methyl group of l-KDF. All amino acid residues in l-KDFDH from *H. huttiense*, except for a Ser-Tyr-Lys motif of the catalytic triad (shadowed in green-yellow), were used for a site-directed mutagenic analysis. (**B**) Phylogenetic tree of the SDR superfamily including l-KDFDH. The number on each branch indicates the bootstrap value. The physiological and/or best substrate(s) for AldDH is in parentheses: l-rhamnose (l-Rha); d-glucose (d-Glu); l-arabinose (l-Ara); d-xylose (d-Xyl); l-fucose (l-Fuc); d-mannose (d-Man); d-fucose (d-Fuc). (**C**) Purification of recombinant (His)_6_-tag proteins. Five micrograms of each purified protein was applied to a 12% (w/v) SDS polyacrylamide gel.

Among metabolic enzymes involved in non-phosphorylating l-fucose pathway, l-KDFDH belongs to the short-chain dehydrogenase/reductase (SDR) superfamily, and has been identified in several bacteria, including *Xanthomonas campestris* (XCC4067)^8^, *Herbaspirillum huttiense* (C785_RS13675)^3^, and *Acidovorax avenae* (ACAV_RS08210)^4^ (Figs. 1D and 2A,B). The SDR superfamily is one of the largest and extensively examined protein groups, consisting of ~ 170,000 primary structures and ~ 1100 three-dimensional structures^9,10^. Despite low sequence identities between different functional subfamilies (approximately 15–30%; also for l-KDFDH), structural frameworks with 250~350 residues in length consist of highly similar α/β folding patterns with a central β-sheet, the so-called Rossmann fold, and the triad of Ser-Tyr-Lys play an important role in catalysis. These common and routine features may limit further analyses of the catalytic mechanism of l-KDFDH since its first discovery in 2006^8^; the crystal structures of FucM^11,12^, FucA^13^, FucB^13^, FucC^8^, FucH^3^, and FucG^14^ have already been elucidated. On the other hand, we recently reported that BDH2, a mammalian homolog with l-KDFDH from bacteria (> 50% of sequence homology), catalyzed the NAD^+^(H)-dependent reversible conversion between 4-oxo-l-proline (4OP) and *cis*-4-hydroxy-l-proline (C4LHyp) (EC 1.1.1.104) (Fig. 1E)^15^. This enzyme was previously suggested to act as a cytosolic type 2 β-d-hydroxybutyrate (β-d-HB) dehydrogenase (EC 1.1.1.30) in the utilization of ketone bodies^16^ or catalyzed the synthesis of 2,3-dihydroxybenzoic acid (2,3-DHBA), a putative mammalian siderophore (EC 1.3.1.28)^17^. The *k*_cat_/*K*_m_ value for 4OP is more than 10^4^-fold higher than that for β-d-HB, and there is a significant structural difference between chain-like l-KDF and cyclic 4OP.

We herein report for the first time the crystal structures of l-KDFDH (from *H. huttiense*) in the ligand-free, l-KDF and l-2,4-DKDF, d-KDP, or l-2,4-DKDF and NADH bound forms at resolutions of 1.27, 1.67, 1.51, and 1.29 Å, respectively. In complexed structures, l-KDF, l-2,4-DKDF, and d-KDP exclusively bound as a α-furanosyl hemiketal form. Although l-KDF exists as a mixture of the acyclic α-keto and furanosyl hemiketal forms in aqueous solution, this was previously based on its chemical properties and only the former was a substrate for the dehydratase and/or aldolase enzymes (Fig. 1D). The C1 carboxyl and α-anomeric C2 hydroxyl groups and O5 oxygen atom of the substrate (and product) were specifically recognized by Arg148, Arg192, and Arg214, which was one of the patterns of arginine ligands that had appeared several times within the l-KDF subfamily in the SDR superfamily in the evolutionary stage.

## Results

### Substrate specificities of l-KDFDH and BDH2

As described in “Introduction”, despite of different physiological role and structural framework of substrate, there is significant sequence homology between bacterial l-KDFDH and mammalian BDH2. Therefore, we initially elucidated substrate specificities between l-KDFDH from *H. huttiense* and BDH2 from human using recombinant enzymes (Table 1).

**Table 1:** Kinetic parameters of l-KDFDH from *H. huttiense* and BDH2 from human.

Enzyme	Substrate		*K*_m_ (mM)	*k*_cat_ (min^–1^)	*k*_cat_/*K*_m_ (min^–1^·mM^–1^)
l-KDFDH	l-KDF	4(*S*)-OH	0.115 ± 0.015^a^	901 ± 19	7900 ± 840
	l-KDGal		0.808 ± 0.073^a^	1190 ± 72	1480 ± 45
	d-KDP		2.58 ± 0.24^b^	1650 ± 122	641 ± 16
	d-KDGlu		6.60 ± 0.55^b^	495 ± 21	75.2 ± 3.3
	l-KDP	4(*R*)-OH	5.51 ± 0.66^b^	202 ± 11	36.9 ± 2.2
	*cis*-4-Hydroxy-l-proline		4.19 ± 0.35^b^	44.5 ± 1.4	10.7 ± 0.6
	*cis*-4-Hydroxy-d-proline		37.8 ± 11.5^b^	0.600 ± 0.145	0.160 ± 0.0009
	*trans*-4-Hydroxy-l-proline		2.26 ± 0.27^b^	0.0992 ± 0.0063	0.0442 ± 0.0027
	*trans*-4-Hydroxy-d-proline		N.D.^c^	N.D	N.D
	β-d-Hydroxybutyrate		1.79 ± 0.57^b^	0.0821 ± 0.0100	0.0477 ± 0.0082
BDH2	l-KDF		0.0322 ± 0.0033^d^	220 ± 11	6860 ± 350
	*cis*-4-Hydroxy-l-proline		0.220 ± 0.061^a^	293 ± 30	1370 ± 220
	β-d-Hydroxybutyrate		18.5 ± 2.9^b^	4.19 ± 0.29	0.228 ± 0.020

Values are the means ± SD, *n* = 3.

^a^Eight different substrate concentrations between 0.1 and 1 mM were used.

^b^Eight different substrate concentrations between 1 and 10 mM were used.

^c^Not assessed due to trace activity.

^d^Eight different substrate concentrations between 0.01 and 0.1 mM were used.

**Figure 3:**
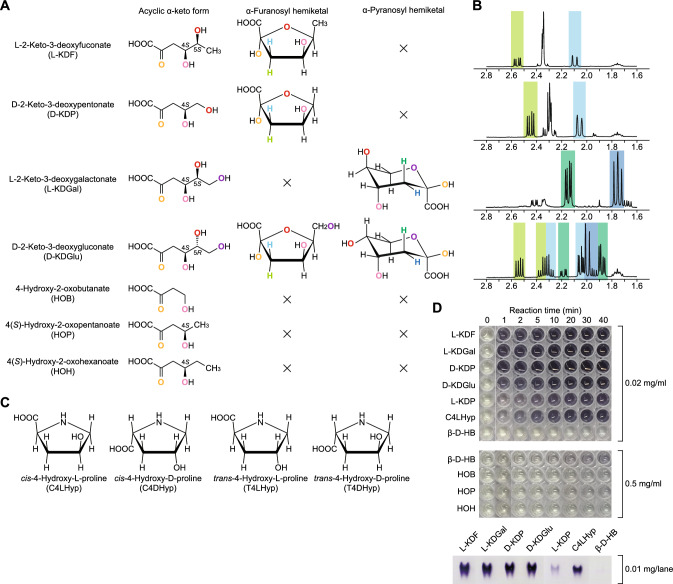
Substrate structure of l-KDFDH. (**A**) Chemical structures of 2-keto-3-deoxysugar acids (α-anomer of hemiketal). Homologous oxygen atoms are indicated in the same color. (**B**) The ^1^H NMR spectrum of 2-keto-3-deoxysugar acid in D_2_O. Characteristic signals derived from two prochiral hydrogens of C3 are indicated in the same color and correspond to (**A**). Whole spectrum is shown in Fig. S9. (**C**) Chemical structures of 4-hydroxyprolines. (**D**) Zymogram staining analysis. The indicated concentration of l-KDFDH from *H. huttiense* was used under the conditions of 10 mM substrate and 10 mM NAD^+^. Alternatively, the enzyme was separated on non-denaturing PAGE, and the gel was then soaked in the same staining solution (lower panel).

The dehydrogenase activity of l-KDFDH was specific for l-KDF, d-KDP, l-2-keto-3-deoxygalactonate (l-KDGal), and d-2-keto-3-deoxygluconate (d-KDGlu) with the 4(*S*)-hydroxyl group (Fig. 3A). Among them, l-KDF was identified as the best substrate, with a *k*_cat_/*K*_m_ value that was 5.3-, 12-, and 105-fold higher than those for l-KDGal, d-KDP, and d-KDGlu, respectively (Table 1). It is known that l-KDFDH physiologically utilizes d-KDP as a substrate in nonphosphorylating d-xylose pathway from bacteria^18^.

Potential 4OP reductase activity in l-KDFDH was estimated by using C4LHyp as a substrate in the reverse reaction (Fig. 1E); BDH2 showed similar specific activity values for 4OP and C4LHyp^15^. C4LHyp was also an active substrate for l-KDFDH, and the *k*_cat_/*K*_m_ (10.7 min^–1^·mM^–1^) value was ~ 220-fold higher than that for β-d-HB (0.0477 min^–1^·mM^–1^), which was caused by the markedly higher *k*_cat_ value (Table 1). On the other hand, the *k*_cat_/*K*_m_ value for l-KDF (7900 min^–1^·mM^–1^) was ~ 760-fold higher than that for C4LHyp, which was caused by a marked decrease in the *K*_m_ value and increase in the *k*_cat_ value. These substrate specificities were also observed in the zymogram staining analysis (Fig. 3D).

Dehydrogenase activity for l-KDF was also detected in BDH2, and the *k*_cat_/*K*_m_ value (6860 min^–1^·mM^–1^) was very similar to that for l-KDFDH from *H. huttiense* (Table 1). On the other hand, the ratio of l-KDF to C4LHyp in the *k*_cat_/*K*_m_ value was only ~ 5, conforming to the physiological substrate of C4LHyp (4OP), which differed from l-KDFDH in bacteria. We assumed that the most likely function of BDH2 as a l-KDFDH is to contribute in the nonphosphorylating l-fucose pathway (route 3; Fig. 1B) in mammals^19^ together with rTSγ^20^ and FAHD1^21^ as (unidentified) FucC and FucG, respectively.

### Overall structural description of l-KDFDH

**Figure 4:**
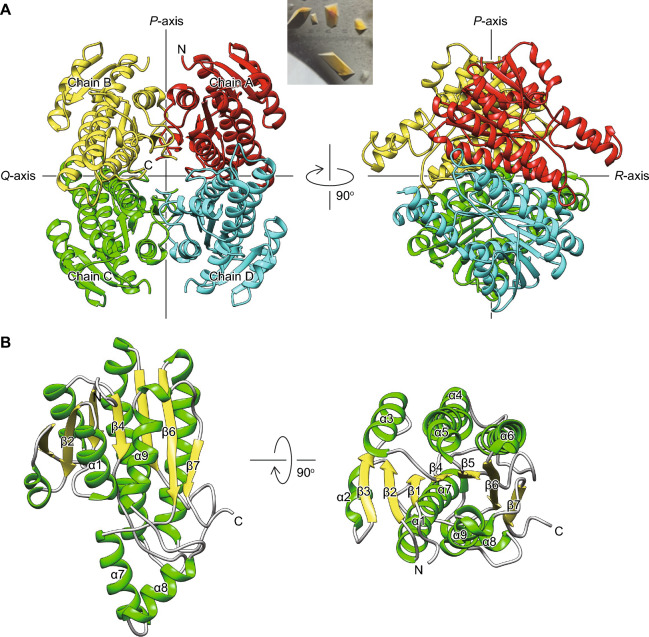
Overall crystal structures of l-KDFDH. (**A**) Ribbon representations of the tetramer viewed along each of the two non-crystallographic two-fold axes. Left and light panels are views along the *R*- and *Q*-axes, respectively. Chains A ~ D are shown in red, yellow, green, and blue, respectively. The inset photo is a crystal of l-KDFDH from *H. huttiense*. (**B**) Ribbon diagram of chain C. α-Helices and β-strands are colored in green and yellow, respectively. A comparison between chains A ~ D is shown in Fig. S2.

l-KDFDH from *H. huttiense* was crystallized under condition No. 22 (Crystal Screen). The crystal structure of the apo-form was elucidated by the molecular replacement method using the structure predicted by *AlphaFold2*^22^ as the search mode, and the model was refined at a resolution of 1.27 Å (PDB ID 8XWK). Data processing and structure refinement statistics are listed in Table 2. The two molecules in the asymmetric unit were essentially identical, being superimposable with a root-mean-square deviation (r.m.s.d.) of 0.112 Å over 254 Cα atoms (Fig. 4A). A tight homotetramer is described as a dimer of dimers with a 222-point group symmetry mediated by three perpendicular twofold axes that are conventionally termed the *P*, *Q*, and *R*-axis^23^.

**Table 2:** Data collection and refinement statistics.

	Apo-form	Sulfate ion and NAD^+^(H) partially bound form	l-KDF and l-2,4-DKDF bound form	d-KDP bound form	l-2,4-DKDF and NADH bound form
PDB ID	8XWK	8Y11	8Y46	8Y4J	8Y4B
Crystallization	No. 22 (crystal screen)	No. 77 (Index HT)	No. 22 (crystal screen)	No. 22 (crystal screen)	No. 22 (crystal screen)
Data collection					
Space group	*P*2_1_2_1_2_1_	*P*2_1_2_1_2_1_	*P*2_1_2_1_2_1_	*P*2_1_2_1_2_1_	*P*2_1_2_1_2_1_
*a, b, c* (Å)	62.76, 112.36, 129.11	84.34, 103.89, 133.06	62.51, 113.05, 129.59	64.79, 112.01, 129.56	60.87, 119.54, 129.29
α, β, γ (º)	90.00, 90.00, 90.00	90.00, 90.00, 90.00	90.00, 90.00, 90.00	90.00, 90.00, 90.00	90.00, 90.00, 90.00
Wavelength (Å)	1.00000	1.00000	1.00000	1.00000	1.00000
Resolution range (Å)	45.00–1.27 (1.29–1.27)	48.39–1.77 (1.80–1.77)	44.99–1.29 (1.31–1.29)	44.71–1.51 (1.54–1.51)	44.315–1.670 (1.70–1.67)
*R*_merge_	0.144 (1.920)	0.315 (4.361)	0.124 (1.271)	0.428 (3.453)	0.385 (2.517)
*R*_meas_	0.148 (1.970)	0.324 (4.473)	0.128 (1.309)	0.445 (3.551)	0.396 (2.580)
CC_1/2_	0.999 (0.821)	0.995 (0.584)	0.998 (0.880)	0.884 (0.791)	0.991 (0.759)
I/σ	17.6 (2.3)	16.0 (1.2)	17.1 (2.5)	10.3 (1.9)	10.1 (2.1)
Completeness (%)	99.8 (99.2)	100.0 (99.9)	100.0 (100.0)	98.3 (99.8)	100.0 (99.9)
Redundancy	19.45 (19.89)	19.84 (20.34)	17.82 (17.49)	18.17 (18.00)	19.57 (20.40)
Refinement					
*R*_work_/*R*_free_	0.1791/0.1995	0.1821/0.2108	0.1794/0.1969	0.1880/0.2104	0.2667/0.3165
No. of atoms					
Protein	7352	7037	7272	7139	7221
Ligands	21	222	63	45	187
Water	1077	464	889	755	393
*B*-factors (Å^2^)					
Protein	18.44	31.79	17.06	25.10	22.12
Ligands	32.77	48.4	24.87	32.15	21.52
Water	29.19	36.79	26.43	32.74	24.81
r.m.s. deviations					
Bond lengths (Å)	0.021	0.017	0.006	0.010	0.007
Bond angles (º)	1.22	1.51	0.88	1.09	0.91

The overall structure and folding topology of l-KDFDH were similar to those of SDR superfamily enzymes^9,10^; r.m.s.d. values of 1.3 ~ 1.5 Å and sequence identities of 23 ~ 35% (Fig. 4B). The closest related structure in the protein data bank (PDB) is BDH2 from human (PDB ID 2AG5); r.m.s.d. of 0.8 Å over 237 Cα atoms with a sequence identity of 54%^16^. However, 2AG5 is the structure in complex with the coenzyme NAD^+^(H). The subunit structure of l-KDFDH mainly consisted of a Rossmann fold dinucleotide cofactor-binding motif^24^, in which a central, twisted β-sheet comprising seven parallel β-strands (β3-β2-β1-β4-β5-β6-β7) is flanked by five helices (α1, α2, α7-α9) on one side and four helices (α3-α6) on the other side. A small domain containing α7 and α8 was slightly separated from the main body of the subunit, and appeared to be rotated by an angle of ~ 30° in chains A, B, and C (Fig. S2). The so-called “closed conformation” is often caused by coenzyme and/or substrate binding in other SDR superfamily enzymes.

**Figure 5:**
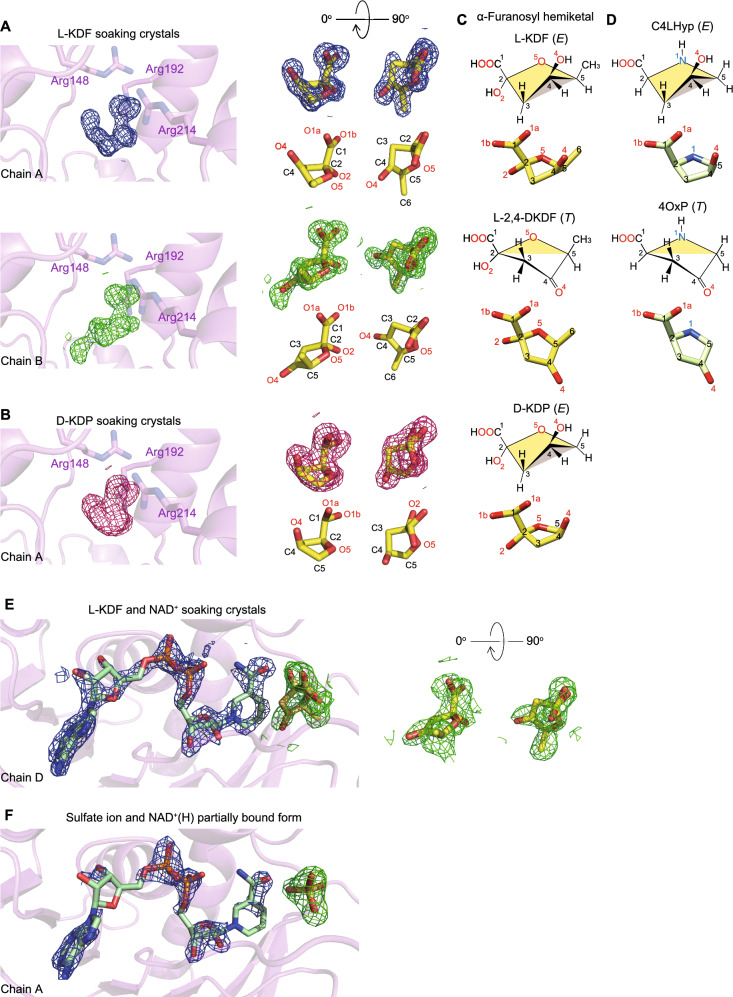
Identification of the substrate structure bound in l-KDFDH. Electron density map of the bound molecules of l-KDF (upper panel in (**A**)), l-2,4-DKDF (lower panel in (**A**)), d-KDP (**B**), l-2,4-DKDF and NADH (**E**), and sulfate ion and NAD^+^(H) (**F**) in 8Y46, 8Y4J, 8Y4B, and 8Y11, respectively. Simulated annealing *mF*_o_-*DF*_c_ difference Fourier maps were calculated by omitting each molecule, and are shown as meshes countered at the 1.0 σ level. Each comparison between chains A ~ D is shown in Figs. S3–S6. In A and B, the middle panel indicates the fitting model of the substrate or product. Schematic structures of α-furanosyl hemiketal (**C**) and pyrrolidine-2-carboxylate (**D**) in the envelope (*E*) and twist (*T*) conformations.

On the other hand, despite no addition of NAD^+^(H) during purification and crystallization, poor electron density for the coenzyme was observed in crystals prepared under different condition No. 77 (Index HT) containing highly concentrated sulfate ion (see Fig. 5F). Although this structure was also refined with reasonable stereochemical quality to a resolution of 1.77 Å (8Y11 in Table 2), the NAD^+^(H) and sulfate ion were located at equivalent positions to those in BDH2 from human (Fig. S3). Therefore, although no electron density for the coenzyme was observed in the apo-from described above, we assumed that the purified l-KDFDH enzyme partially contained NAD^+^(H).

### Structure in complex with l-KDF, l-2,4-DKDF, or d-KDP

To elucidate the structure of l-KDFDH in complex with substrates, l-KDF, d-KDP, l-KDGal, d-KDGlu, C4LHyp, or 2,3-DHBA was soaked in crystals of the apo-form, prepared under condition No. 22 (Crystal Screen). Among them, the crystal structures in complex with l-KDF and d-KDP were successfully determined to resolutions of 1.29 and 1.51 Å, respectively (8Y46 and 8Y4J in Table 2). In the *F*_o_-*F*_c_ omit map, increased electron density was unambiguously observed inside the active pocket of chains A, B, and D for l-KDF (Fig. 5A and Fig. S4) and chains A, C, and D for d-KDP (Fig. 5B and Fig. S5). These electron density maps clearly contained the structural framework of the α-furanosyl hemiketal, but not the acyclic α-keto form (Figs. 1D and 3A). Among them, one of the ring atoms (C4), except chain B for l-KDF, was located outside and above the plane defined by the four other atoms (C2-C3-C5-O5) (Fig. 5C). On the other hand, two of the ring atoms of chain B for l-KDF (C3 and C4) were located outside the plane defined by the three other atoms (C2-C5-O5), one above this plane (C3) and the other (C4) below it. These features corresponded to the envelope and twist conformations of furanose, respectively (referred to as species *E* and *T*)^25^.

We assumed that one of these molecular species (species *T*) was an enzyme product for the following reasons. NAD^+^ was partially bound in crystals in the apo-form (Fig. 5F), by which the enzyme reaction may occur in a crystallization droplet during soaking in a solution containing a substrate and cryoprotectant. The preference for l-KDF over d-KDP as a substrate may explain why species *T* was only observed in l-KDF and not d-KDP (Table 1). Furthermore, C4LHyp and 4OP, deposited in the PDB (HZP and DPL, respectively), also formed the envelope and twist conformations and superimposed well on species *E* and *T*, respectively (Fig. 5D). Based on these insights, we assigned species *E* in chains A and D for l-KDF and chains A, C, and D for d-KDP, and species *T* in chain B for l-KDF as l-KDF, d-KDP, and l-2,4-DKDF, respectively.

**Figure 6:**
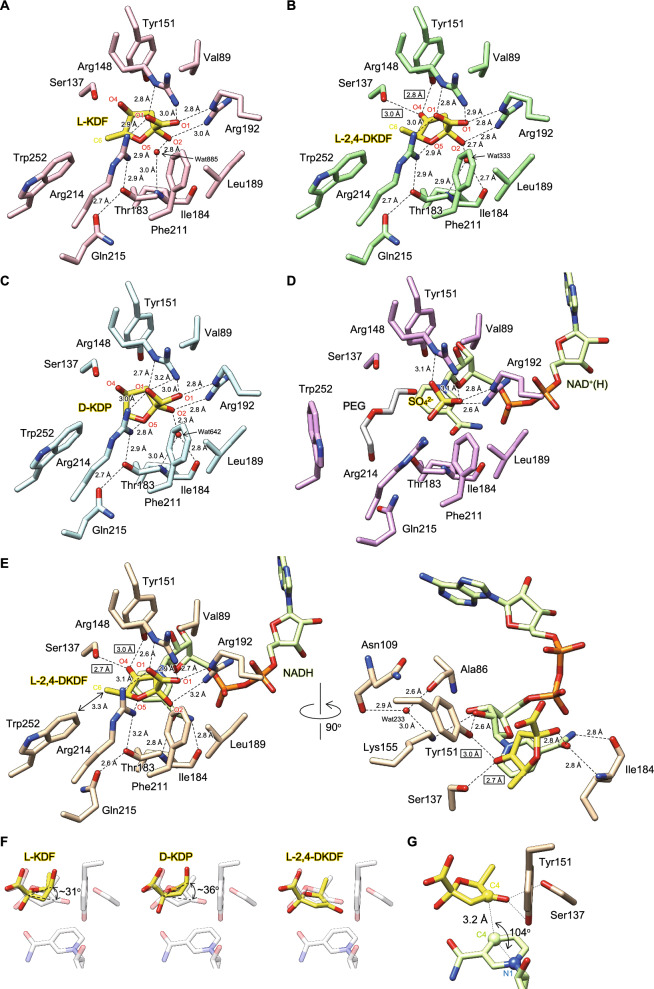
Interaction mode of the substrate and product (and NADH). Crystals of the apo-form were soaked in reservoir solution containing l-KDF (**A** and **B**), d-KDP (**C**), sulfate ion and NAD^+^(H) (**D**), or l-KDF and NAD^+^ (**E**). Each electron density is shown in Fig. 5A, B and E. Hydrogen bonds are shown as black broken lines, and those between the O4 oxygen atom of l-2,4-DKDF and amino acid residues are in the box. (**F**) The superposition of l-KDP, d-KDP, and l-2,4-DKDF in the absence (yellow) and presence (transparent) of NADH. (**G**) Position between the nicotinamide ring and l-2,4-DKDF.

The carboxyl group of bound l-KDF, d-KDP, and l-2,4-DKDF commonly formed salt bridges with the side chains of Arg148, Arg192, and Arg214 from three directions (Fig. 6A–C), and was located at an equivalent position to sulfate ion in the sulfate ion and NAD^+^(H) partially bound form (Fig. 6D). In the ligand-free and PEG-bound forms, these side chains (particularly Arg192 and Arg214) were frequency disordered (Figs. S2–S5). The α-anomeric C2 hydroxyl group made hydrogen bonds with a water molecule and the side chain of Arg192, and the former further interacted with the main chain nitrogen and/or oxygen atom(s) of Ile184. The O5 atom of the furanose ring interacted with the side chain of Arg214, which further formed a hydrogen bond network with the side chains of Thr183 and Gln215. Importantly, the C4 keto oxygen atom of l-2,4-DKDF (but not the C4 hydroxyl group of l-KDF and d-KDP) interacted with the hydroxyl side chains of Ser137 and Tyr151 (boxes in Fig. 6B). Among the active site pockets containing these residues, each alanine mutant of Val89, Arg148, Thr183, Leu189, Arg192, Phe211, Arg214, Gln215, and Trp252 was constructed; most of these mutants were tightly conserved in other l-KDFDH enzymes (Fig. 2A). Their *k*_cat_/*K*_m_ values for l-KDF (except for the Q215A mutant) were markedly reduced by 2 ~ 3 orders of magnitude from the wild-type (WT) enzyme (the R214A mutant was inactive), conforming to the structural insights obtained (Table 3).

**Table 3:** Kinetic parameters for l-KDF by mutants of l-KDFDH from *H. huttiense*.

Enzymes	*K*_m_ (mM)	*k*_cat_ (min^–1^)	*k*_cat_/*K*_m_ (min^–1^·mM^–1^)
WT	0.115 ± 0.015^a^	901 ± 19	7900 ± 840
V89A	25.9 ± 3.8^b^	152 ± 17	5.89 ± 0.34
R148A	0.171 ± 0.008^a^	1.60 ± 0.03	9.33 ± 0.27
T183A	0.380 ± 0.057	2.00 ± 0.09	5.32 ± 0.54
L189A	4.45 ± 0.16^b^	12.6 ± 0.4	2.84 ± 0.01
R192A	1.63 ± 0.54^a^	18.3 ± 4.8	11.4 ± 0.7
F211A	9.33 ± 0.90^b^	12.3 ± 1.1	1.32 ± 0.01
R214A	N.D.^c^	N.D	N.D
Q215A	0.594 ± 0.123^a^	122 ± 17	206 ± 13
W252A	1.40 + 0.24^a^	55.8 ± 7.0	39.9 ± 1.80
W252F	0.494 ± 0.136^a^	409 ± 52	847 ± 110
W252M	0.219 ± 0.006^a^	397 ± 10	1800 ± 5

Values are the means ± SD, *n* = 3.

^a^Eight different substrate concentrations between 0.1 and 1 mM were used.

^b^Eight different substrate concentrations between 1 and 10 mM were used.

^c^No preparation of recombinant protein.

### Structure in complex with NADH and l-2,4-DKDF

To elucidate the reaction mechanism in more detail, we attempted to determine the structure in complex with the substrate and coenzyme, the Michaelis ternary complex. To achieve this, crystals in the apo-form, prepared under condition No. 22 (Crystal Screen), were soaked in solution containing NAD^+^ and l-KDF for a few minutes. The increased (continuous) electron density of not only the coenzyme (in all chains), but also other molecule was unambiguously observed inside the active pocket of chain D only, and the latter was identical to the product (Fig. 5E and Fig. S6). Therefore, this structure was refined to resolution of 1.67 Å as the NADH and l-2,4-DKDF bound form (8Y4B in Table 2).

There were two differences from the structure in complex with (only) l-KDF or l-2,4-DKDF. The α-anomeric C2 hydroxyl group of l-2,4-DKDF only interacted with the side chain of Arg192, and the main chain nitrogen and oxygen atoms of Ile184 formed hydrogen bonds with the O7 and N7 atoms of the nicotinamide ring, respectively (Fig. 6E). Furthermore, l-2,4-DKDF was rotated by an angle of ~ 31° to the nicotinamide ring from the position of l-KDF in the only substrate bound form (~ 36° for d-KDP); there was no positional difference of l-2,4-DKDF between the only product bound form (Fig. 6F). We assumed that this binding position of l-2,4-DKDF was also homologous to l-KDF in the Michaelis ternary complex with l-KDF and NAD^+^, by which the C4 hydroxyl group of l-KDF formed hydrogen bonds to the hydroxyl side chains of Ser137 and Tyr151, as well as other SDR superfamily enzymes. Among the nearby amino residues from l-2,4-DKDF and NADH, Ser137-Tyr151-Lys155, corresponding to a motif of the catalytic triad, and Ala94 and Asn117 were completely conserved in the SDR superfamily including other l-KDFDH enzymes (Fig. 2A) ^9,10^. The distance between C4 of l-2,4-DKDF and C4* of the nicotinamide ring of NADH and the angle of N1-C4*-C4 (3.2 Å and 104°, respectively) were consistent with those obtained from various structural analyses of the hydride-transferring enzyme in complex with NAD(P)^+^ (Fig. 6G). In the putative catalytic mechanism, the C4 hydroxyl group of l-KDF was deprotonated by Tyr151 as a general base catalyst, with the concurrent transfer of the hydride ion to NAD^+^, and the p*K*_a_ value of its hydroxyl group was reduced by Lysl55, leading to the stabilization of the tyrosinate anion at physiological pH. Based on these insights, we concluded that l-KDFDH utilized the α-furanosyl hemiketal of l-KDF as a substrate.

### Coenzyme binding mode

**Figure 7:**
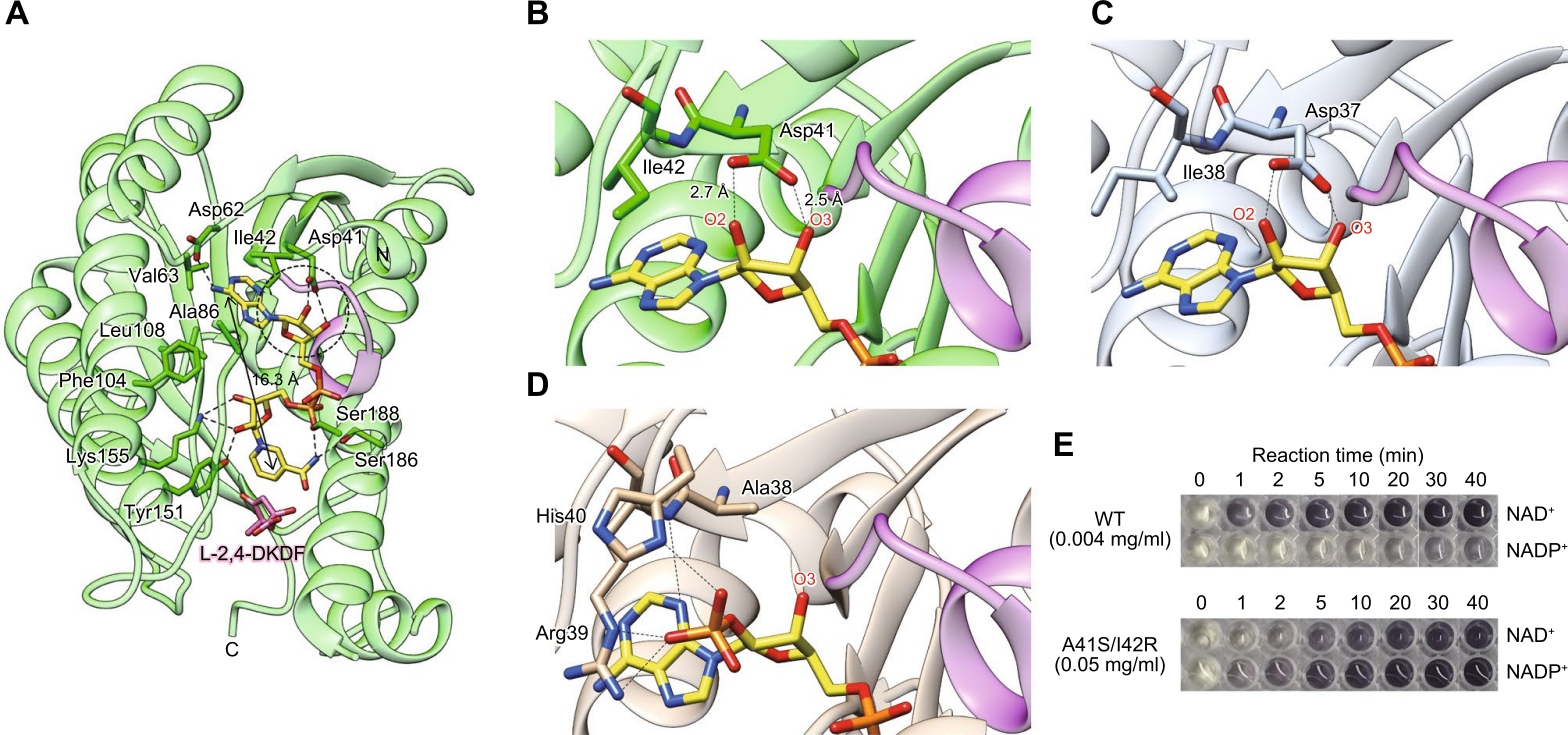
Interaction mode of coenzymes. (**A**) Overall structure in complex with coenzyme and product (8Y4B). NADH and l-2,4-DKDF molecules are shown as stick models colored in yellow and pink, respectively. The residues responsible for NADH recognition are shown as stick models. Hydrogen bonds are shown as black broken lines. The purple-colored region corresponds to the characteristic sequence, [Gly/Ala]-X_3_-Gly-X-Gly, in a typical dinucleotide binding Rossmann fold motif. Region with broken line corresponds to (**B**). Recognition of the 2’- and 3’-hydroxyl groups of NADH by l-KDFDH from *H. huttiense* (**B**) and BDH2 from human (2AG5) (**C**), and the 2’-phosphate group of NADP^+^ by FucA from *B. multivorans* (4GVX) (**D**). (**E**) Zymogram staining analysis. The indicated concentration of l-KDFDH from *H. huttiense* was used under the conditions of 10 mM l-KDF and 10 mM NAD(P)^+^.

In the structures of 8Y4B and 8Y11, NADH commonly bound at the C-terminal edge of the seven-stranded parallel β-sheet in an extended conformation; the distance between C6 of the adenine ring and C2 of the nicotinamide ring is 16.3 Å (Fig. 7A). The region of Ala17-Ala18-Ala19-Gln20-Gly21-Ile22-Gly23, located between the end of β1 and the start of α1 (purple), corresponds to the extended consensus sequence for coordinating the cofactor pyrophosphate in the Rossman fold. The adenine ring of NADH binds in a hydrophobic pocket on the enzyme surface formed by the side chains of Ile42, Val63, Ala86, Phe104, and Leu108. A hydrogen bond is formed between N1 of adenine and the Asp62 side chain. Furthermore, the 2’- and 3’-hydroxyl groups of the adenine ribose form double hydrogen bonds with the OD1 and OD2 atoms of the side chain of Asp41 (Fig. 7B), as well as the structure of BDH2 in complex with NAD^+^(H) (Fig. 7C).

A pair of aspartates and the nearby hydrophobic residues, corresponding to Asp41-Ile42 in l-KDFDH from *H. huttiense*, were strictly conserved among other l-KDFDH enzymes (Fig. 2A). In NADP^+^-dependent enzymes, this aspartate residue is commonly replaced by a smaller and uncharged residue, such as glycine, alanine, and serine, accompanied by the concurrent presence of an arginine residue that forms a positive-binding pocket for the 2’-phosphate group of NADP^+^^10^, such as the pair of Ala38-Arg39 in FucA from *Burkholderia multivorans*^13^ (Fig. 7D). In fact, a large-scale phylogenetic analysis using the Protein-BLAST program revealed that several (putative) l-KDFDH enzymes possessed the pair of Asp-Arg or Ser-Arg at these positions (IEX19_RS15075 and PSMK_RS04265 in Fig. 2A, respectively), according to which the I42R and D41S/I42R mutants of l-KDFDH from *H. huttiense* were designed. Among them, the *k*_cat_/*K*_m_ value for the D41S/I42R mutant for NAD^+^ markedly decreased (up to 2400-fold) due to a reduction in *k*_cat_, while that for NADP^+^ increased by ~ 470-fold due to a reduction in the *K*_m_ value and a marked increase in the *k*_cat_ value, by which the ratio of NADP^+^ to NAD^+^ of the D41S/I42R mutant changed from 0.00004 to 46, compared with the WT enzyme (Table 4). Similar results were obtained in the zymogram staining analysis (Fig. 7E). Collectively, these suggested the coenzyme specificity of l-KDFDH is common to other NAD^+^(H)-dependent SDR enzymes.

**Table 4:** Kinetic parameters of WT and mutants of l-KDFDH from *H. huttiense* for NAD(P)^+^.

Enzyme	Coenzyme	*K*_m_ (mM)	*k*_cat_ (min^–1^)	*k*_cat_/*K*_m_ (min^–1^·mM^–1^)	NADP^+^, NAD^+^
WT	NAD^+^	0.0302 ± 0.003^a^	1750 ± 78	58,300 ± 3520	0.00004
	NADP^+^	1.77 ± 0.30^b^	4.07 ± 0.60	2.31 ± 0.06	
I42R	NAD^+^	0.157 ± 0.010^c^	128 ± 1	824 ± 50	0.009
	NADP^+^	1.27 ± 0.10^b^	8.08 ± 0.45	7.14 ± 0.20	
D41S/I42R	NAD^+^	0.142 ± 0.029^c^	3.34 ± 0.50	23.8 ± 3.5	46
	NADP^+^	0.200 ± 0.004^c^	216 ± 4	1083 ± 5	

Values are the means ± SD, *n* = 3.

^a^Eight different substrate concentrations between 0.01 and 0.1 mM were used.

^b^Eight different substrate concentrations between 1 and 10 mM were used.

^c^Eight different substrate concentrations between 0.1 and 1 mM were used.

### Phylogenetic analysis of l-KDFDH

The Arg148, Arg192, and Arg214 cluster in l-KDFDH from *H. huttiense*, which recognized the C1 carboxyl group of l-KDF, is the most characteristic motif among SDR superfamily enzymes, and was completely conserved in other functional l-KDFDH enzymes with > 50% sequence similarity (referred to as type 1) (Fig. 2A,B). Although Thr183 did not form a direct interaction with l-KDF, the homologous threonine residue was also frequently found in type 1 l-KDFDH. The R148A, T183A, and R192A mutants of l-KDFDH had very low *k*_cat_/*K*_m_ values and R214A was inactive (Table 3), strongly suggesting their important roles in catalysis (a pattern of R-T-R-R).

On the other hand, a large-scale phylogenetic analysis revealed several hypothetical proteins with low sequence similarity (37 ~ 39%) to type 1 l-KDFDH within the same subfamily branch in the SDR superfamily (Fig. 2A,B). Their patterns of (putative) arginine ligands were classified into types 2 ~ 4 (patterns of R-T-Y-R, R-R-R-T, and R-R-F-T), and the other amino acid residues in the active site pocket were not conserved, except for the catalytic triad of Ser-Tyr-Lys. Therefore, to elucidate the function of type 2 ~ 4 hypothetical proteins, we selected PEH01S_RS21285, PSMK_RS04265, and SMI01S_RS12625 from *Paenibacillus ehimensis* NBRC 15659, *Phycisphaera mikurensis* NBRC 102666, *Sphingobacterium mizutaii* NBRC 14946, respectively, and prepared each recombinant protein (Fig. 2C). They exhibited strict NAD^+^-dependent dehydrogenase activity for l-KDF, conforming to the putative involvement of the nonphosphorylating l-fucose pathway based on the gene context on the bacterial genome (Fig. 1C).

No significant differences were observed in *k*_cat_/*K*_m_ values between type 1 and types 2 and 4 l-KDFDH (7900, 2520, and 2731 mM^–1^·min^–1^, respectively; Table 5). Type 2 l-KDFDH was phylogenetically close to a SDR protein from *Rhizobium phaseoli* in the PDB (4DQX; not yet published) (Fig. 2A,B). Although this structure was the apo-form, only two arginine ligands, Arg170 and Arg231, appeared to recognize the C1 carboxyl and C2 hydroxyl groups and O5 oxygen atom of l-2,4-DKDF (l-KDF) (Fig. S7A). Furthermore, in the models of types 3 and 4 l-KDFDH constructed using *AlphaFold2*^22^, Arg198 and Arg183, respectively, appeared to be structurally homologous to the third arginine ligand in type 1 l-KDFDH (Fig. S7B,C). Based on these structural insights, we attempted to artificially modify arginine ligands in l-KDFDH. The F192R, R183T/T211R, and R183T/F192R/T211R mutants of SMI01S_RS12625 (type 4), corresponding to types 3, 2, and 1, respectively, were active enzymes, and the R183T/T211R mutant maintained significant activity (Table 5). Collectively, these results indicate that arginine ligands (and also the nearby residues) had been independently (convergently) optimized between types 1 ~ 4 l-KDFDH enzymes.

**Table 5:** Comparison of kinetic parameters for l-KDF between types 1 ~ 4 l-KDFDH enzymes.

Enzymes	Source	Type	Pattern^a^	*K*_m_ (mM)	*k*_cat_ (min^–1^)	*k*_cat_/*K*_m_ (min^–1^·mM^–1^)
C785_RS13675	NBRC 102521	1	R-T-R-R	0.115 ± 0.015^b^	901 ± 19	7900 ± 840
PEH01S_RS21285	NBRC 15659	2	R-T-Y-R	0.529 ± 0.021^b^	1330 ± 23	2520 ± 62
PSMK_RS04265	NBRC 102666	3	R-R-R-T	6.08 ± 0.15^c^	697 ± 13	115 ± 1
SMI01S_RS12625	NBRC 14946					
WT		4	R-R-F-T	0.154 ± 0.004^b^	442 ± 8	2730 ± 77
F192R		3	R-R-R-T	0.326 ± 0.007^b^	0.280 ± 0.005	0.860 ± 0.016
R183T/T211R		2	R-T-F-R	1.18 ± 0.10^b^	116 ± 7	98.8 ± 3.8
R183T/F192R/T211R		1	R-T-R-R	0.135 ± 0.025^b^	0.0947 ± 0.0045	0.714 ± 0.108

Values are the means ± SD, *n* = 3.

^a^Four amino acid residues at equivalent positions to Arg148, Thr183, Arg192, and Arg214 in C785_RS13675 (l-KDFDH from *H. huttiense* NBRC 102521).

^b^Eight different substrate concentrations between 0.1 and 1 mM were used.

^c^Eight different substrate concentrations between 1 and 10 mM were used.

## Discussion

### Hemiketal structure as a substrate

2-Keto-3-deoxysugar acids, including l-KDF, exist as a mixture of the acyclic α-keto form and furanosyl/pyranosyl hemiketal in aqueous solution (Fig. 3A); the ^1^H NMR spectrum of the sample dissolved in D_2_O highlights characteristic signals derived from two prochiral hydrogens at the C3 position of the furanosyl/pyranosyl hemiketal (Fig. 3B). However, it has been believed that this is simply derived from the physicochemical property, and only the acyclic α-keto form is a substrate for the dehydratase^3,26–28^ and/or aldolase enzymes^2,29–32^, whose reaction intermediate is a covalent Schiff base formed between the carbonyl C2 atom and a lysine residue^2,27–30,32^ or octahedral coordination with both the C1 carboxyl and the C2 oxo groups, carboxyl residues, and water molecule(s)^3,26,31^.

In the crystal structure of d-KDGlu kinase from *Sulfolobus solfataricus* in complex with substrate(s) (2VAR), the electron density of substrate is assigned as both the acyclic form and β-furanosyl hemiketal of d-KDGlu, although it is likely that the former is better positioned for phosphoryl transfer^33^. In this regard, to completely avoid the “misassignment” of electron density of substrate in the complexed structure(s) of l-KDFDH, we further synthesized 4(*S*)-hydroxy-2-oxopentanoate (HOP) and 4(*S*)-hydroxy-2-oxohexanoate (HOH), and 4-hydroxy-2-oxobutanate (HOB). Among them, HOP and HOH correspond to d-KDP and l-KDF substituted the C5 hydroxyl groups to hydrogen atom, respectively, and C4 hydroxyl group of HOB is achiral (Fig. 3A). Commonly, their acyclic α-keto forms possess 4(*S*)-hydroxyl group, but not cyclize as a hemiketal in principle. As the result, l-KDFDH showed no activity toward these compounds (Fig. 3D), suggesting that only hemiketal is substrate. To the best of our knowledge, this is the first example for the physiological role of the hemiketal.

### Structural insights into substrate specificity

Although the soaking of C4LHyp (and 2,3-DHBA) in crystals of the apo-form was not successful, the recognition of l-KDF as a hemiketal structure (but not the acyclic α-keto form) may explain why l-KDFDH co-utilizes cyclic C4LHyp as a substrate. Among four 4-hydroxyproline isomers, only C4LHyp possessed the same configuration as those of C2 and C4 as the α-furanosyl hemiketal of l-KDF and was an active substrate (Fig. 3C). C4LHyp fits into the electron density map for d-KDP, except for the C2 hydroxyl group (data not shown). In the other words, the lack of its interaction with the side chain of Arg192 (Fig. 6C) may be responsible for the ~ 60-fold lower *k*_cat_/*K*_m_ value for C4LHyp than for d-KDP (Table 1).

Among four active substrates, the hemiketal structures of l-KDF and d-KDP were the furanose form only (Fig. 3A). The side chain of Trp252 was the closest to the C6 methyl group (a distance of 3.3 Å; Fig. 6E), and l-KDFDH enzymes possessed the tightly conserved hydrophobic residue at an equivalent position (most commonly tryptophan, methionine, phenylalanine, and leucine; Fig. 2A). Therefore, the W252F and W252M mutants were further designed together with the W252A mutant. The *k*_cat_/*K*_m_ values for l-KDF were reduced by an order of magnitude from WT > W252F≈W252M > W252A (Table 3), indicating the importance of the hydrophobic interaction. This effect was specific for l-KDF, which may be due to the ~ 12-fold higher *k*_cat_/*K*_m_ value for l-KDF than for d-KDP (Table 1). Another possibility is the smaller percentage of the hemiketal of d-KDP in aqueous solution (~ 70%) than l-KDF (~ 100%), l-KDGal (> 99%), and d-KDGal (~ 97%)^34^.

Before starting this study, we were interested in why l-KDFDH exhibited moderate activity towards l-KDP with the 4(*R*)-hydroxyl group (Table 1), which was also detectable in the zymogram staining analysis (Fig. 3D). Therefore, we constructed a docking model by fitting l-KDP in an electron density map for d-KDP (Fig. S8). When l-KDP binds at a homologous position to d-KDF as a α-furanosyl hemiketal, the transfer of the C4 proton to NAD^+^ as a hydride ion (H^-^) is impossible due to the opposite direction. On the other hand, complete rotation by an angle of 180°, with concurrent binding as a “β-furanosyl hemiketal”, enables this proton to turn to the nicotinamide ring and interact between the C1 carboxyl and C2 hydroxyl groups and three arginine ligands; however, the hydrogen bond between the O5 oxygen atom and Arg214 is eliminated. This positional rearrangement may be allowed for l-KDP only without the protruding chemical groups of the furanose ring. To confirm this hypothesis, we are currently attempting to elucidate the crystal structure in complex with l-KDP.

### Molecular evolution of l-KDFDH in SDR superfamily

**Figure 8:**
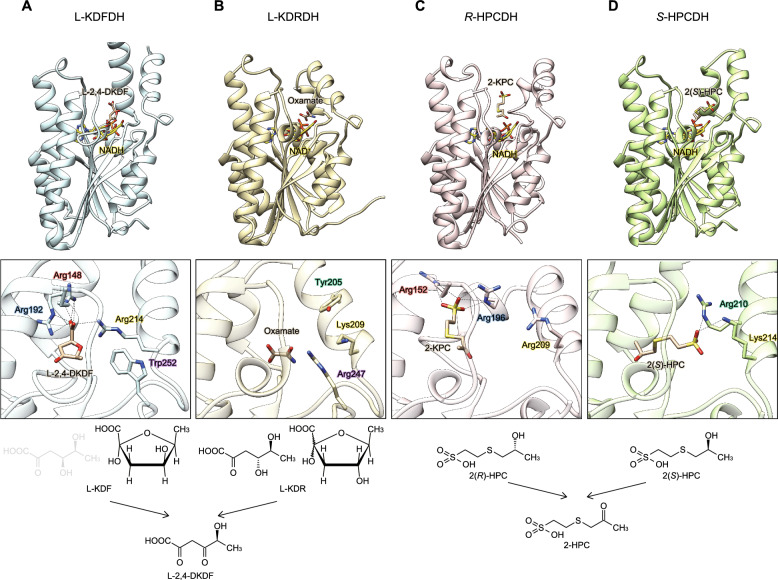
Comparisons of overall structures and active sites between l-KDFDH (**A**), putative l-KDRDH (**B**), *R*-HPCDH (**C**), and *S*-HPCDH (**D**). Amino acid residues in the same color are sequentially homologous to each other. Lower panels indicate a schematic reaction. The substrate conformation for l-KDRDH is unclear.

A large-scale phylogenetic analysis also revealed that types 2 ~ 4 l-KDFDH enzymes were close to hydroxypropyl-CoM dehydrogenase (EC 1.1.1.269; HPC), which catalyzes the oxidation of (*R*)- or (*S*)-enantiomer of 2-HPC to the achiral product 2-ketopropyl-CoM (2-KPC); *R*-HPCDH (2CFC) and *S*-HPCDH (4ITU), respectively^35,36^ (Fig. 2B). Arg152, Arg196, and Arg209 in *R*-HPCDH were not only structurally, but also sequentially homologous to the three arginine ligands of type 1 l-KDFDH enzymes, with Arg152 and Arg196 interacting with a sulfonate moiety of 2(*R*)-HPC (Fig. 8A,C,D).

As described in “Introduction”, l-KDR 4-dehydrogenase (l-KDRDH) showed the opposite stereoselectivity at the C4 hydroxyl group to l-KDFDH (Fig. S1). Although the crystal structure of l-KDRDH was unavailable, the closest related structure in the PDB, the hypothetical SDR protein from *Burkholderia phymatum* (6D9Y; not yet published), showed 51 and 54% sequence identities with the l-KDRDH enzymes from *H. huttiense* (C785_RS20555) and *Sphingomonas* sp. (SKA58_RS01175), respectively (Fig. 2B)^3,5^. In this putative l-KDRDH, two basic residues (Lys209 and Arg247) were located within the same side of the active site pocket as the two ligands for the sulfonate moiety of 2(*S*)-HPC in *S*-HPCDH (Arg205 and Lys208); the two lysine residues were sequentially homologous to each other (Fig. 8A,B,D).

Collectively, these indicate possibilities that the binding mode for the negatively charged chemical group are derived from the same origin between l-KDFDH and *R*-HPCDH, and the “switch” required for chiral discrimination between l-KDFDH and l-KDRDH is due to the differential placement of positively charged residues that recognize the C1 carboxyl group of 2-keto-3-deoxysugar acid substrates, similar to* R*-HPCDH and *S*-HPCDH. We are currently attempting to elucidate the crystal structure of l-KDRDH in complex with substrates.

## Conclusion

The trigger for this study was to understand how l-KDFDH can recognize “chain-like” l-KDF and “cyclic” C4LHyp (4OP) with different structural frameworks. A crystallographic analysis revealed that l-KDFDH utilized l-KDF as α-furanosyl hemiketal, but not the acyclic α-keto form. The acyclic α-keto form was previously considered to function as a substrate for enzymes; however, its presence in aquatic solutions is very low. The C1 carboxyl group (and α-anomeric hydroxyl group) was recognized by three unique arginine residues, the numbers and/or positions of which had been independently optimized between known and more ancestral l-KDFDH enzymes. The present results provide insights into the molecular evolution of the diversified SDR superfamily.

## Methods

### Bacterial strains

*H. huttiense* NBRC 102406, *P. ehimensis* NBRC 15659, *P. mikurensis* NBRC 102666, and *S. mizutaii* NBRC 14946 were purchased from the National Institute of Technology and Evaluation (NITE) (Chiba, Japan). Genomic DNA was prepared using a DNeasy Tissue Kit (Qiagen).

### Gene cloning and protein overexpression and purification

The primer sequences used in the present study are shown in Table S1. Target genes (C785_RS13675 (l-KDFDH from *H. huttiense*), PEH01S_RS21285, PSMK_RS04265, and SMI01S_RS12625) were amplified by PCR from genomic DNA using KOD One DNA polymerase (Toyobo) and introduced into the *Bam*HI-*Hin*dIII site in pQE-80L (Qiagen), a plasmid vector that encodes an N-terminal (His)_6_-tag added to the expressed protein, in order to obtain each pQE-based expression plasmid. Recombinant *E. coli* cells harboring the constructed plasmids were grown at 37 °C to a turbidity of 0.6 at 600 nm in LB medium containing ampicillin (50 mg/l). After the addition of 1 mM isopropyl-β-d-thiogalactopyranoside, the culture was further grown at 37 °C for 6 h to induce the expression of the respective (His)_6_-tagged protein. Cells were harvested and resuspended in Buffer A (50 mM sodium phosphate buffer (pH 8.0) containing 300 mM NaCl and 10 mM imidazole). Cells were then disrupted by sonication and the solution was centrifuged. The supernatant was loaded onto a Ni-NTA Superflow column (Qiagen). The column was washed with Buffer B (50 mM sodium phosphate buffer (pH 8.0) containing 300 mM NaCl, 10% (v/v) glycerol, and 25 mM imidazole). The enzymes were then eluted with Buffer C (pH 8.0, Buffer B containing 250 mM instead of 25 mM imidazole). Regarding the crystallization of l-KDFDH from *H. huttiense*, the proteins were further loaded onto a HiLoad 16/600 Superdex 200 pg column (GE Healthcare) equilibrated with Buffer D (20 mM Tris–HCl (pH 8.0) containing 150 mM NaCl). The main single-peak fractions were collected and concentrated by ultrafiltration with Amicon Ultra-15 (Millipore). Recombinant (His)_6_-tagged BDH2 from human was expressed in *E. coli* cells and purified, as previously described^15^.

### Site-directed mutagenesis

Several mutants of l-KDFDH enzymes from *H. huttiense* and *S. mizutaii* were constructed using a PCR-based method with the mutated sense and antisense primers (Table S1) and each pQE-based expression plasmid as a template. Mutant proteins were expressed and purified by the same procedures as the WT enzyme.

### Synthesis of 2-keto-3-deoxysugar acid substrates

All sugar acids, except for l-threonate (Tokyo Chemical Industry, Japan), were prepared by hypoiodite-in-methanol oxidization from the corresponding sugars as K^+^ or Ba^2+^ salts^46^. The solution containing sugar acids was applied to the column of an AG^®^ 1-X8 Resin (200–400 mesh, formate form) (Bio-Rad). The column was washed thoroughly with water and developed with a gradient of 0–1 M formic acid. Fractions containing sugar acids were combined and lyophilized to yield the corresponding lactone sugars. Sugar acids were obtained by the base hydrolysis of the lactone sugar according to the method of Yew et al.^8^.

l-KDF, l-KDGal, d-KDP, d-KDGlu, l-KDP, and HOB were enzymatically synthesized from l-fuconate, l-galactonate, d-xylonate, d-gluconate, l-arabinonate, and l-threonate using bacterial dehydratases for d-arabinonate (for l-KDF and l-KDGal), and l-arabinonate (for d-KDP, d-KDGlu, l-KDP, and HOB) (Supplementary discussion)^2–4^. Briefly, the reaction mixture (100 ml) consisted of 50 mM Hepes–NaOH buffer (pH 7.2), 10 mM sugar acid, and 1 mM MgCl_2_. After the addition of ~ 50 mg purified sugar acid dehydratase, the mixture was left at 30 °C overnight. The solution was filtered and then applied to an AG^®^ 1-X8 Resin column followed by a 0–1 M (for l-KDF, l-KDGal, d-KDP, d-KDGlu, and l-KDP) or 0–6 M (for HOB) gradient of formic acid, and fractions containing 2-keto-3-deoxygugar acid (detected using the semicarbazide method)^37^ were combined, lyophilized, and dissolved. A stock solution (100 mM) was prepared by neutralization (~ pH 7.0) with 1 N NaOH. NMR data were recorded using an ECS400 spectrometer (JEOL RESONANCE Inc., Tokyo, Japan) (Fig. S11).

HOP and HOH were enzymatically synthesized through the aldol-condensation reaction by 4-hydroxy-2-oxoheptanedioate aldolase (HpaI)^38^. To achieve this, HpaI gene was amplified by PCR using the genomic DNA of *E. coli* BL21(DE3) and synthetic DNA primers (Table S1), and then introduced into the *Bam*HI-*Hin*dIII site in pQE-80L. The (His)_6_-tagged recombinant HpaI protein was expressed in *E. coli* cells and purified through the same procedures as l-KDFDH. Briefly, 1 g of sodium pyruvate and 5 ml of pure acetaldehyde or propionaldehyde were mixed with ~ 50 mg purified HpaI protein in a total of 50 ml of 50 mM Hepes–NaOH buffer (pH 7.2) containing 0.2 mM CoCl_2_. After incubation at 30 °C overnight, the cobalt was removed by incubation with Chelex 100 column (BioRad), and excess aldehyde was evaporated using prior to lyophilization. This procedure gives preparation of a racemic mixture of 4(*R*)- and 4(*S*)-HOP or HOH in approximately equal concentrations.

### Enzyme assay

Dehydrogenase activity in l-KDFDH and BDH2 was measured using a continuous spectrophotometric assay at 340 nm at 30 °C in 50 mM Tris–HCl buffer (pH 8.0) containing 10 mM substrate and 1.5 mM NAD^+^. One unit of all enzyme activities refers to 1 μmol NADH produced/min. *K*_m_ and *k*_cat_ values were calculated by a Lineweaver–Burk plot.

### Zymogram staining analysis

The purified enzyme was incubated in staining solution consisting of 50 mM Tris–HCl (pH 8.0), 0.25 mM nitroblue tetrazolium, 0.06 mM phenazine methosulfate, 10 mM substrate, and 10 mM NAD(P)^+^ at room temperature several times. Dehydrogenase activity appeared as a dark color in solution. Alternatively, the purified enzyme (10 μg) was separated on non-denaturing PAGE with 8% gel at 4 °C. The gel was then soaked in 10 ml of the same staining solution (containing NAD^+^ as a coenzyme) at room temperature for 15 min. The dehydrogenase activity appeared as a dark band.

### Crystallization and X-ray crystallography

All crystallization trials were performed at 20 °C using the sitting-drop vapor diffusion method. In this method, drops (0.5 μl) of ~ 20 mg/ml l-KDFDH from *H. huttiense* in Buffer D were mixed with equal amounts of reservoir solution (as follows) and equilibrated against 70 μl of the same reservoir solution by vapor diffusion; 100 mM Tris–HCl (pH 8.5), 200 mM sodium acetate, and 30% (w/v) PEG 4000. Crystals of the holo-form of l-KDFDH from *H. huttiense* were obtained by soaking the crystals of the apo-form in the same reservoir solution supplemented with a substrate (l-KDF or d-KDP) and/or coenzyme (NAD^+^ or NADH). All crystals were directly mounted onto a nylon loop and kept in a stream of nitrogen gas at 100 K during data collection; the concentration of PEG 400 in the reservoir solution was sufficient as a cryoprotectant.

Diffraction data were collected with the PILATUS 6 M detector of BL45XU at SPring-8 (Hyogo, Japan) as well as the processed ZOO system and XDS^39–41^. The structure was elucidated using the molecular-replacement pipeline program BALBES^42^ with the structure predicted by *AlphaFold2*^22^ as the search model. Further model building for structures was manually performed with COOT^43^ and crystallographic refinement with PHENIX^44^. Detailed data collection and processing statistics are shown in Table 2.

### Sequence comparison

Protein sequences were analyzed using the Protein-BLAST and Clustal W programs distributed by the Kyoto Encyclopedia of Genes and Genomes of Japan (KEGG, https://www.genome.jp/kegg/)^45^. The phylogenetic tree was produced using the TreeView 1.6.6. program.

### Supplementary Information

Supplementary Information.

## Data Availability

The coordinates and structural factors of l-KDFDH from *H. huttiense* have been deposited in the Protein Data Bank under the accession codes 8XWK (the apo-form), 8Y11 (the sulfate ion and NAD^+^(H) partially bound form), 8Y46 (the l-KDF and l-2,4-DKDF bound form), 8Y4J (the d-KDP bound form), and 8Y4B (the l-2,4-DKDF and NADH bound form). All other data are available from the corresponding authors upon reasonable request.

## Abbreviations

l-KDF: l-2-keto-3-deoxyfuconate
l-2,4-DKDF: l-2,4-diketo-3-deoxyfuconate
l-KDFDH: l-KDF 4-dehydrogenase
l-KDR: l-2-keto-3-deoxyrhamnonate
SDR: Short-chain dehydrogenase/reductase
C4LHyp: *cis*-4-Hydroxy-l-proline
4OP: 4-Oxo-l-proline
β-d-HB: β-d-hydroxybutyrate
d-KDP: d-2-keto-3-deoxypentonate
l-KDGal: l-2-keto-3-deoxygalactonate
d-KDGlu: d-2-keto-3-deoxygluconate
HOP: 4-Hydroxy-2-oxopentanoate
HOH: 4-Hydroxy-2-oxohexanoate
HOB: 4-Hydroxy-2-oxobutanate
HPCDH: Hydroxypropyl-CoM dehydrogenase
l-KDRDH: l-KDR 4-dehydrogenase
