# Supplementary material for: Crystal structure of l-2-keto-3-deoxyfuconate 4-dehydrogenase reveals a unique binding mode as a α-furanosyl hemiketal of substrates
Source: Sci Rep. 2024 Jun 25;14:14602. doi: 10.1038/s41598-024-65627-8 (PMC11199699; doi:10.1038/s41598-024-65627-8)
Supplement: Supplementary file 1 — Supplementary Information. [file 41598_2024_65627_MOESM1_ESM.pdf]

Supplementary information

Crystal structure of L-2-keto-3-deoxyfuconate 4-dehydrogenase reveals a unique binding mode as a α-furanosyl hemiketal of substrates

Miyu Akagashi[1#], Seiya Watanabe[1,2,3#*], Sebastian Kwiatkowski[4], Jakub Drozak[4], Shin-ichi Terawaki[5], Yasunori Watanabe[6]

[1] Department of Bioscience, Graduate School of Agriculture, Ehime University, Matsuyama, Ehime, Japan
[2] Faculty of Agriculture, Ehime University, Matsuyama, Ehime, Japan
[3] Center for Marine Environmental Studies (CMES), Ehime University, Matsuyama, Ehime, Japan
[4] Department of Metabolic Regulation, Institute of Biochemistry, Faculty of Biology, University of Warsaw, Warsaw, Poland
[5] Division of Structure Analysis of Protein Complex, Proteo-Science Center (PROS), Ehime University, Matsuyama, Ehime, Japan
[6] Faculty of Science, Yamagata University, 1-4-12 Kojirakawa-machi, Yamagata, Yamagata 990-8560, Japan
[#] Contributed equally

[*]For correspondence. E-mail: irab@agr.ehime-u.ac.jp; Tel./Fax: +81-89-946-9848

This PDF file includes:
Table S1
Figures S1 to S9

Table S1. Primers used in the present study

Primer	Sequence
Cloning of the L-KDFDH gene from *H. huttiense* into pQE-80L[1]	
P1 (BamHI)	5′-catggaTCCGCATCCACTGGCCGCCTCGCCG-3′
P2 (HindIII)	5′-attaagctTTAGTTGGACCACCCACCATCAATC-3′
Cloning of the L-KDFDH gene from *P. ehimensis* into pQE-80L[1]	
P3 (BamHI)	5′-catggatccAGATTGCACAATAAGGTAACGCTCATTAC-3′
P4 (HindIII)	5′-attaagcTTATGCGTTTTTGCCGAAGACGACG-3′
Cloning of the L-KDFDH gene from *P. mikurensis* into pQE-80L[1]	
P5 (BglII)	5′-catagatctGAAGTCGACGCGCTGGCCCATCTGAC-3′
P6 (HindIII)	5′-attaagctTCAGGAGCGGCCGGCGCTCCAACCC-3′
Cloning of the L-KDFDH gene from *S. mizutaii* into pQE-80L[1]	
P7 (BglII)	5′-catagatctAGGCTAAAAGATAAAGTAGCGATTGTG-3′
P8 (HindIII)	5′-attaagcttTAGTTATTCAGCTTAACAAATCCTCCG-3′
Cloning of the HpaI gene from *E. coli* into pQE-80L[1]	
P11 (BglII)	5′-catagatctGAAAACAGTTTTAAAGCGGCGCTGAAAG-3′
P12 (HindIII)	5′-attaagcTTAATACACGCCGGGCTTCACGGCGG-3′
Site-directed mutagenesis in the L-KDFDH gene from *H. huttiense*[2]	
P13 (V89A-F)	5'-ggctacgcagcggccggcaacattctggag-3'
P14 (V89A-R)	5'-ggccgctgcgtagcccgcgcagttgaagag-3'
P15 (R148A-F)	5'-gccaatgcctttgcctacggtgcctccaag-3'
P16 (R148A-R)	5'-ggcaaaggcattggccacgcccttcacgct-3'
P17 (T183A-F)	5'-ccgggcgccattgaatcgccctcgctgaac-3'
P18 (T183A-R)	5'-ttcaatggcgcccgggcagatcgcattgca-3'
P19 (L189A-F)	5'-ccctcggcgaaccagcgcatcagcacgcaa-3'
P20 (L189A-R)	5'-ctggttcgccgagggcgattcaatggtgcc-3'
P21 (R192A-F)	5'-aaccaggccatcagcacgcaagccaaggag-3'
P22 (R192A-R)	5'-gctgatggcctggttcagcgagggcgattc-3'
P23 (F211A-F)	5'-gccgccgccgtggcacgccagcccatgggc-3'
P24 (F211A-R)	5'-tgccacggcggcggcgcggacttcgtcttc-3'
P25 (R214A-F)	5'-gtggcagcccagcccatgggccgcatcggc-3'
P26 (R214A-R)	5'-gggctgggctgccacgaaggcggcgcggac-3'
P27 (Q215A-F)	5'-gcacgcgcgcccatgggccgcatcggcaag-3'
P28 (Q215A-R)	5'-catgggcgcgcgtgccacgaaggcggcgcg-3'
P29 (W252A-F)	5'-ggtggggcgtccaactaaagcttaattagc-3'
P30 (W252A-R)	5'-gttggacgccccaccatcaatcatgtggat-3'
P31 (W252F-F)	5'-ggtgggttctccaactaaagcttaattagc-3'
P32 (W252F-R)	5'-gttggagaacccaccatcaatcatgtggat-3'
P33 (W252M-F)	5'-ggtgggatgtccaactaaagcttaattagc-3'
P34 (W252M-R)	5'-Gttggacatcccaccatcaatcatgtggat-3'
P35 (W252R-F)	5'-ggtgggcggtccaactaaagcttaattagc-3'
P36 (W252R-R)	5'-gttggaccgcccaccatcaatcatgtggat-3'
P37 (T183R-F)	5'-ccgggcaggattgaatcgccctcgctgaac-3'
P38 (T183R-R)	5'-ttcaatcctgcccgggcagatcgcattgca-3'
P39 (I42R-R)	5'-accgatcgcagcaagacgcatctggaggag-3'
P40 (I42R-R)	5'-cttgctgcgatcggtagcgatcacgcgcgc-3'
P41 (D41A/I42R-F)	5'-gctaccgctcgcagcaagacgcatctggag-3'
P42 (D41A/I42R-R)	5'-cttgctgcgagcggtagcgatcacgcgcgc-3'
P43 (D41S/I42R-F)	5'-gctaccagtcgcagcaagacgcatctggag-3'

P44 (D41S/I42R-R)	5'-cttgctgcgactggtagcgatcacgcgcgc-3'
Site-directed mutagenesis in the L-KDFDH gene from *S. mizutaii*[2]	
P45 (R183T-F)	5'-ccagcaacagtgcatacgccttttgttgat-3'
P46 (R183T-R)	5'-atgcactgttgctggggagatggaatttga-3'
P47 (F192R-F)	5'-gatggacgcatcgccaaaaactatccaggt-3'
P48 (F192R-R)	5'-ggcgatgcgtccatcaacaaaaggcgtatg-3'
P49 (T211R-F)	5'-tctaaacgccaacctatcggacggatggca-3'
P50 (T211R-R)	5'-aggttggcgtttagaaagtttttcaaacat-3'
[1] Lower case letters indicate additional bases for introducing the underlined digestion sites of restriction enzymes in parentheses.	
[2] F and R indicate forward and reverse primers, respectively. Underlining indicates mutated regions.	

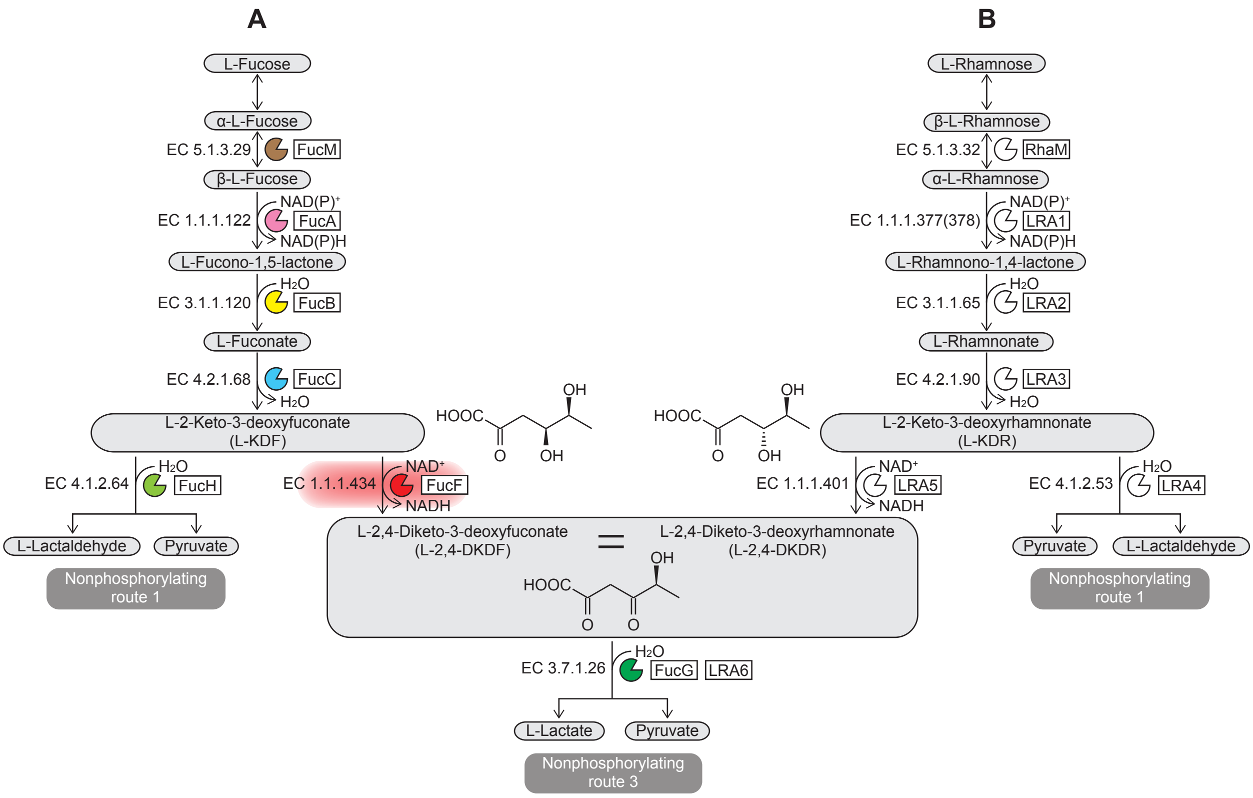

Figure S1. Nonphosphorylating pathways of L-fucose (A) and L-rhamnose (B) metabolism.
L-Fucose and L-rhamnose are commonly converted to the L-2-keto-3-deoxysugar acid intermediates, L-2-keto-3-deoxyfuconate fuconate (L-KDF) and L-2-keto-3-deoxyrhamnonate (L-KDR), respectively, by the consecutive actions of mutarotase, dehydrogenase, lactonase, and dehydratase. One of the metabolic fates of L-KDF and L-KDR is the aldol-cleavage reaction by different aldolases (FucH and LRA4), by which pyruvate and L-lactaldehyde are produced (route 1). In alternative route 3, L-LDF and L-KDR intermediates lose chirality at C4 by the different dehydrogenases (FucF and LRA5), by which the same intermediate as L-2,4-diketo-3-deoxyrhamnonate (L-2,4-diketo-3-deoxyrhamnonate) is produced. Metabolic products are commonly hydrolyzed to pyruvate and L-lactate by hydrolases (FucG or LRA6), which are phylogenetically close each other.

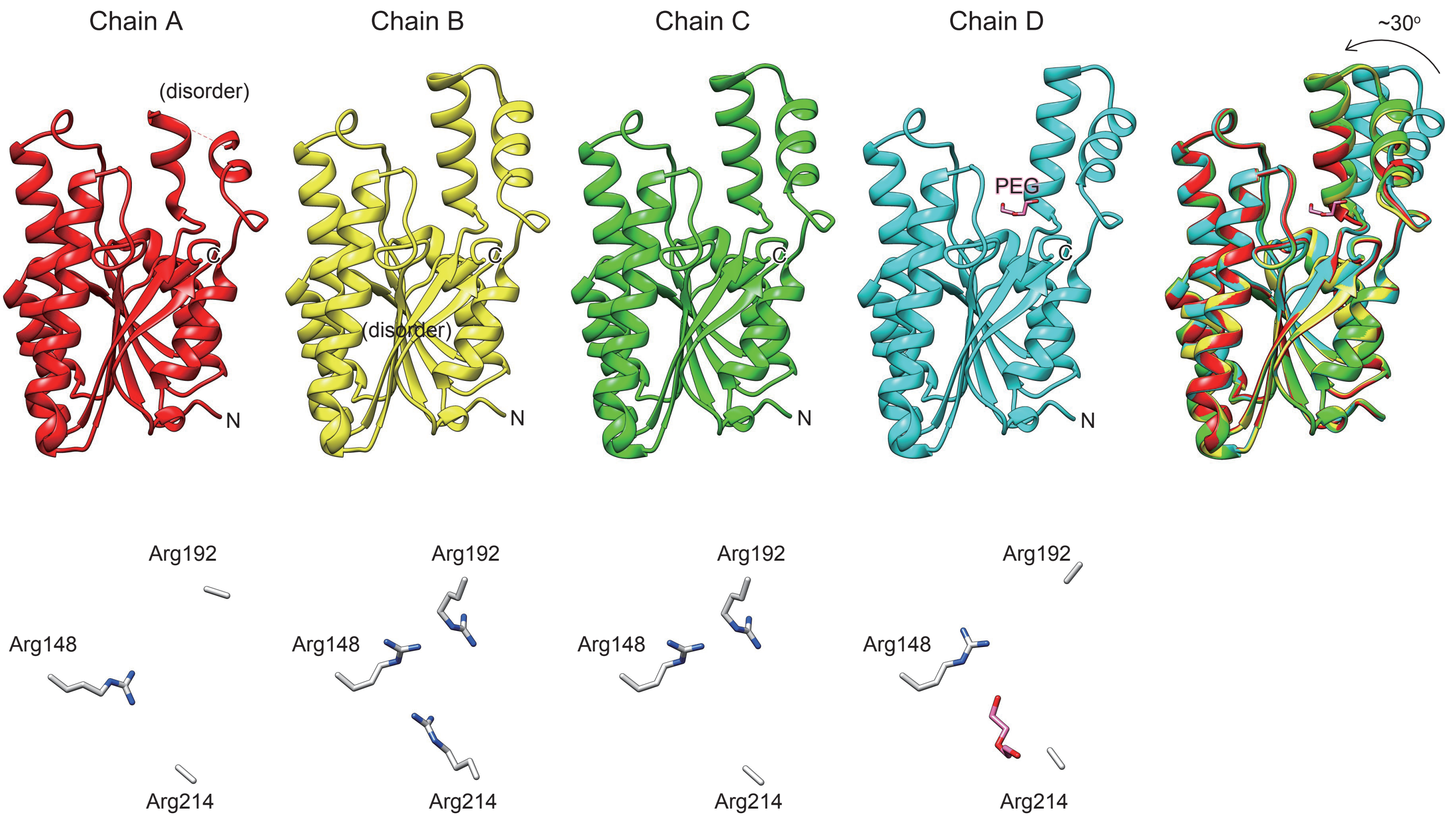

Figure S2. Comparison of the overall structure (upper panel) and three arginine ligands (lower panel) between chains A~D in the ligand-free form of L-KDFDH from *H. huttiense*.
Chain C was used as the apo-from in the text. The r.m.s.d. values of chain C with chains A, B, and D are 0.469, 0.286, and 0.406 Å, respectively.

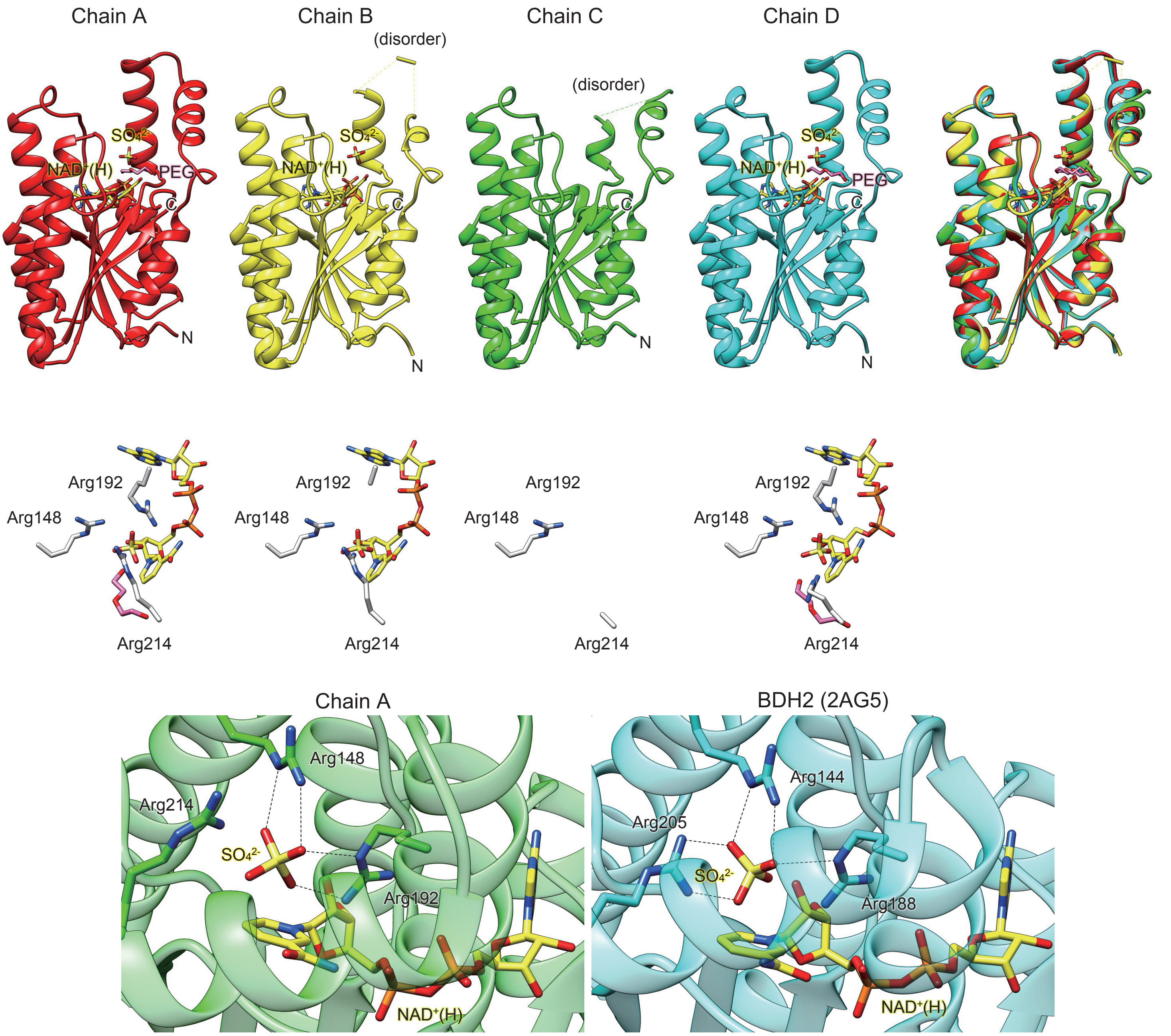

Figure S3. Comparison of the overall structure (upper panel) and three arginine ligands (lower panel) between chains A~D in the sulfate ion and NAD$^+$(H) partially bound form of L-KDFDH from *H. huttiense*.
The r.m.s.d. values of chain A with chains B, C, and D are 0.217, 0.435, and 0.185 Å, respectively. Lower panels indicate comparable interaction mode of sulfate ion and NAD$^+$(H) between L-KDFDH (left) and BDH2 from human (right).

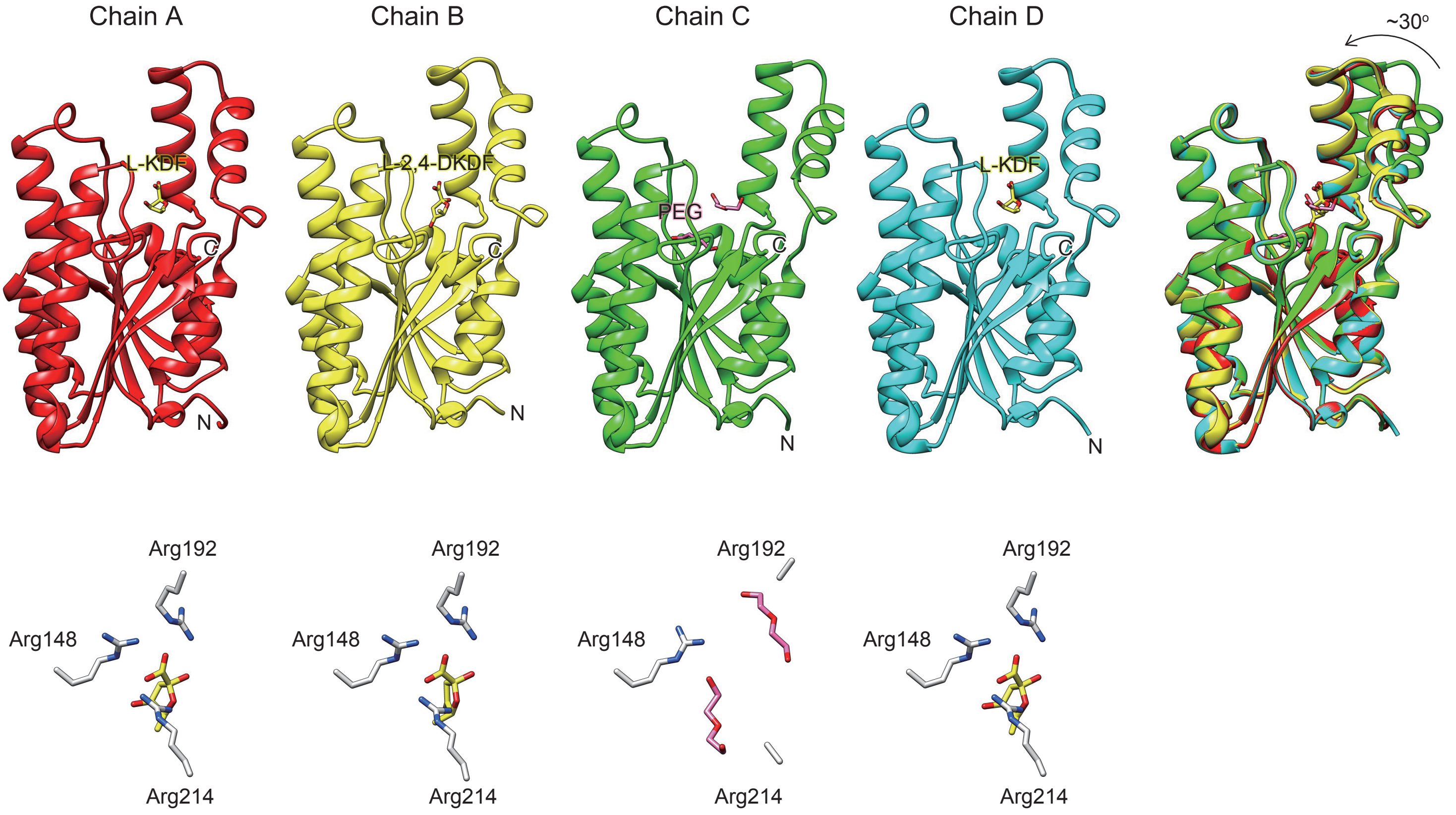

Figure S4. Comparison of the overall structure (upper panel) and three arginine ligands (lower panel) between chains A~D in L-KDF and the L-2,4-LDKDF bound form of L-KDFDH from *H. huttiense*.
Chains A and B were used as the L-KDF or L-2,4-DKDF bound forms, respectively, in the text. The r.m.s.d. values of chain A with chains B, C, and D are 0.377, 0.336, and 0.673 Å, respectively.

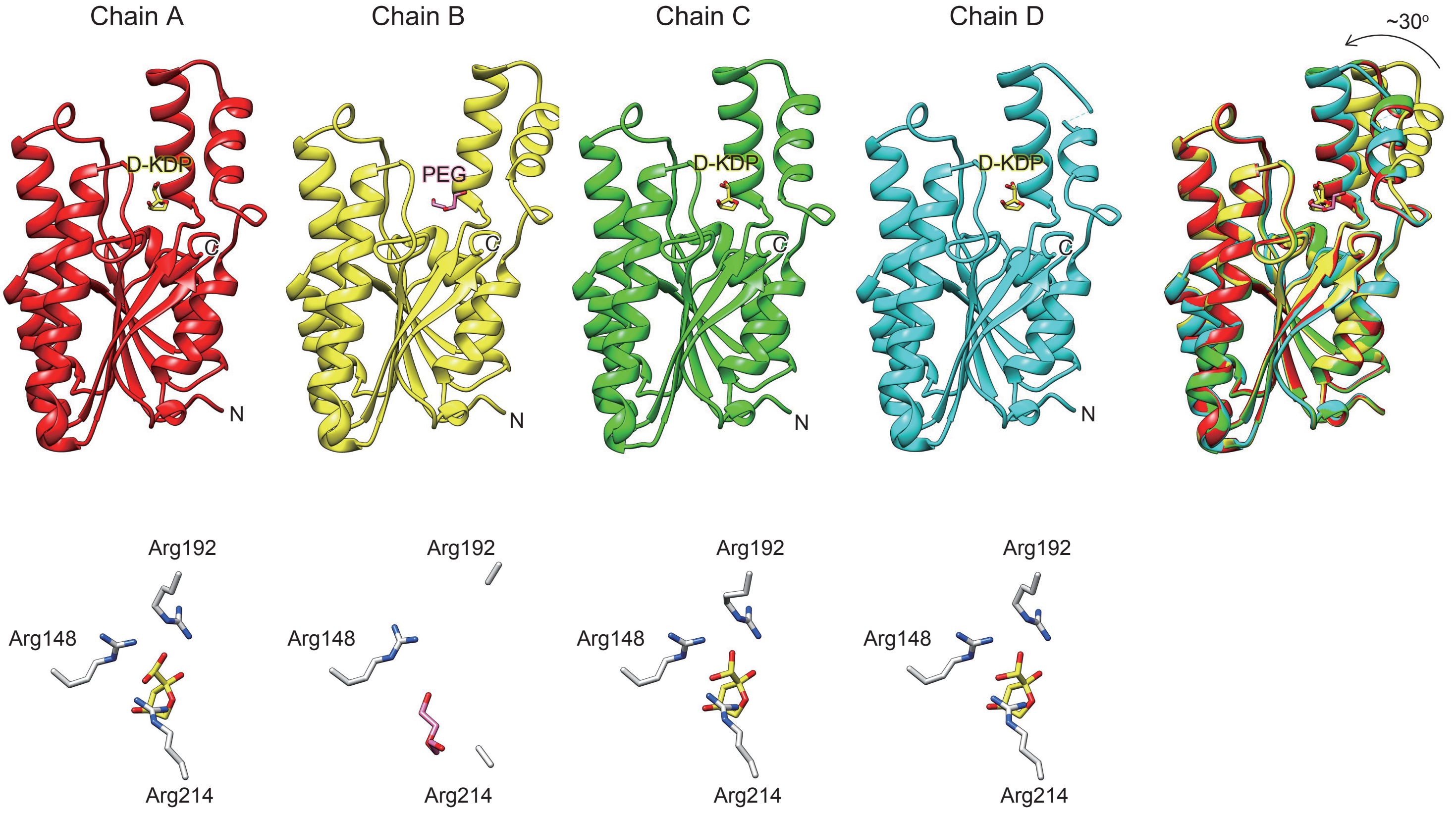

Figure S5. Comparison of the overall structure (upper panel) and three arginine ligands (lower panel) between chains A~D in the D-KDP bound form of L-KDFDH from *H. huttiense*.
Chain C was used as the D-KDP bound form in the text. The r.m.s.d. values of chain C with chains A, B, and D are 0.846, 1.005, and 0.863 Å, respectively.

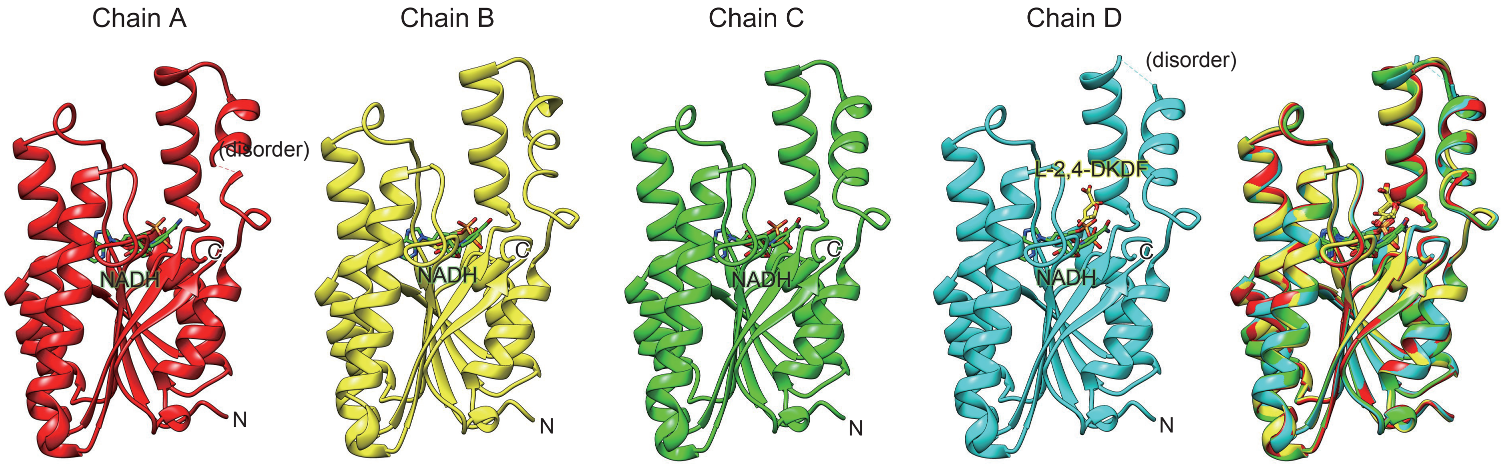

Figure S6. Comparison of the overall structure (upper panel) between chains A~D in NADH and the L-2,4-LDKDF bound form of L-KDFDH from *H. huttiense*.
Chain D was used as the L-2,4-DKDF and NADH bound form in the text. The r.m.s.d. values of chain D with chains A, B, and C are 0.348, 0.801, and 0.345 Å, respectively.

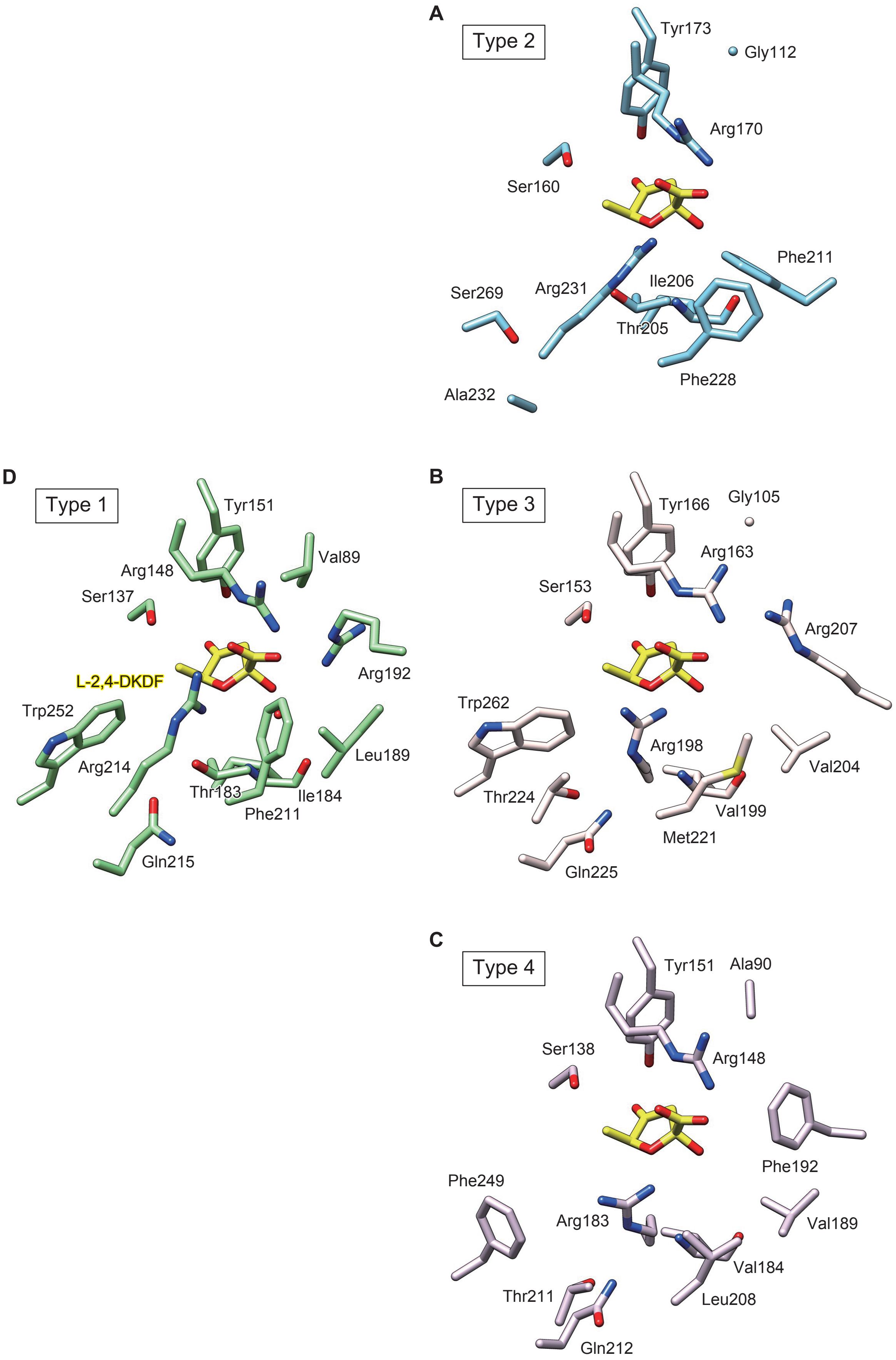

Figure S7. Superposition on structures of type 2 (A), type 3 (B), and type 4 (C) (putative) L-KDFDH on the L-2,4-DKDF bound form of L-KDFDH from *H. huttiense* (D).

(A) A SDR protein from *Rhizobium phaseoli* (4DQX). Models of PSMK_RS04265 (B) and SMI01S_RS12625 (C) constructed using *AlphaFold2*.

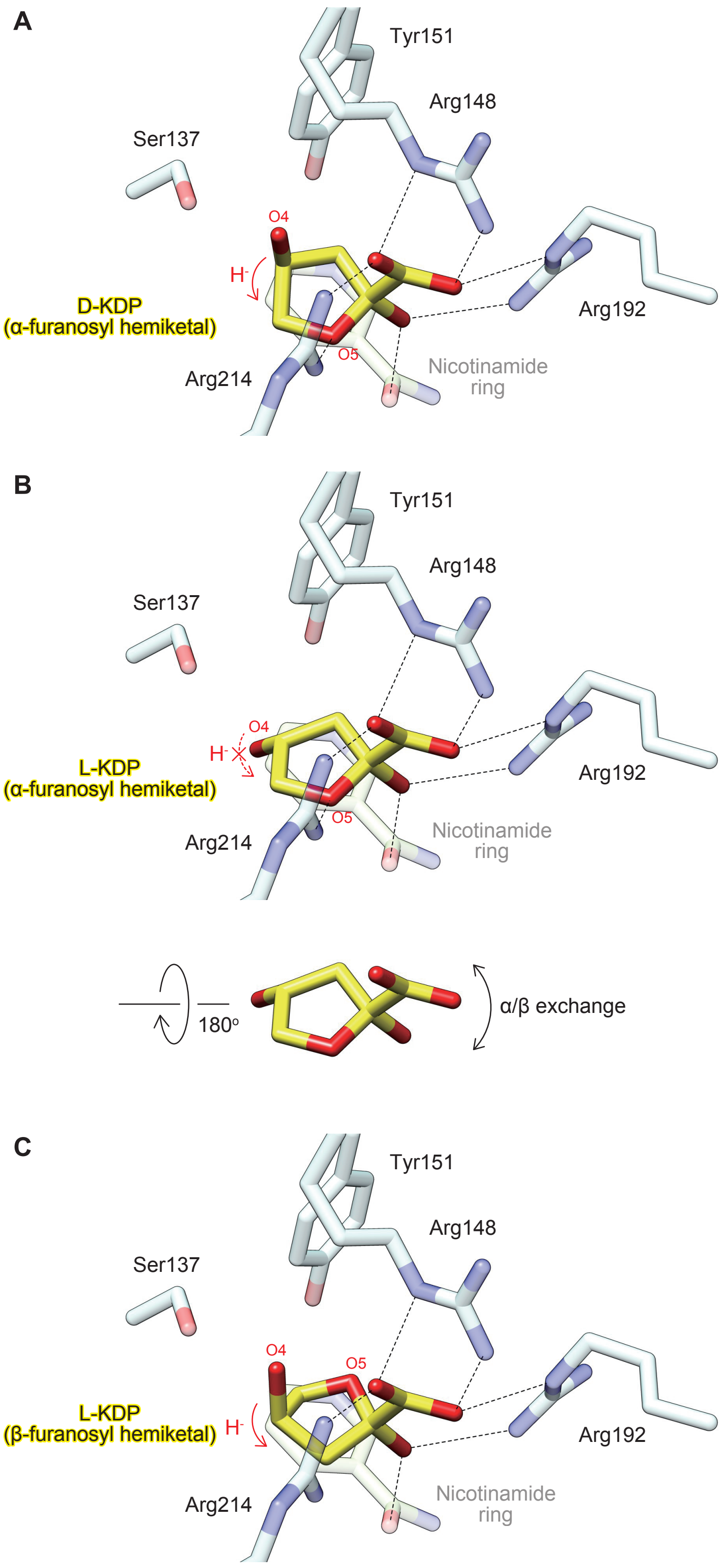

Figure S8. Hypothetical binding mode of L-KDP.
(A) Binding of α-furanosyl hemiketal of D-KDP in the crystal structure of the D-KDP bound form. (B) Model of fitting α-furanosyl hemiketal of L-KDP in the electron density map of D-KDP. The transfer of a hydride ion (H^-) to NAD^+ is impossible. (C) Model of fitting the β-furanosyl hemiketal of L-KDP in the electron density map of D-KDP.

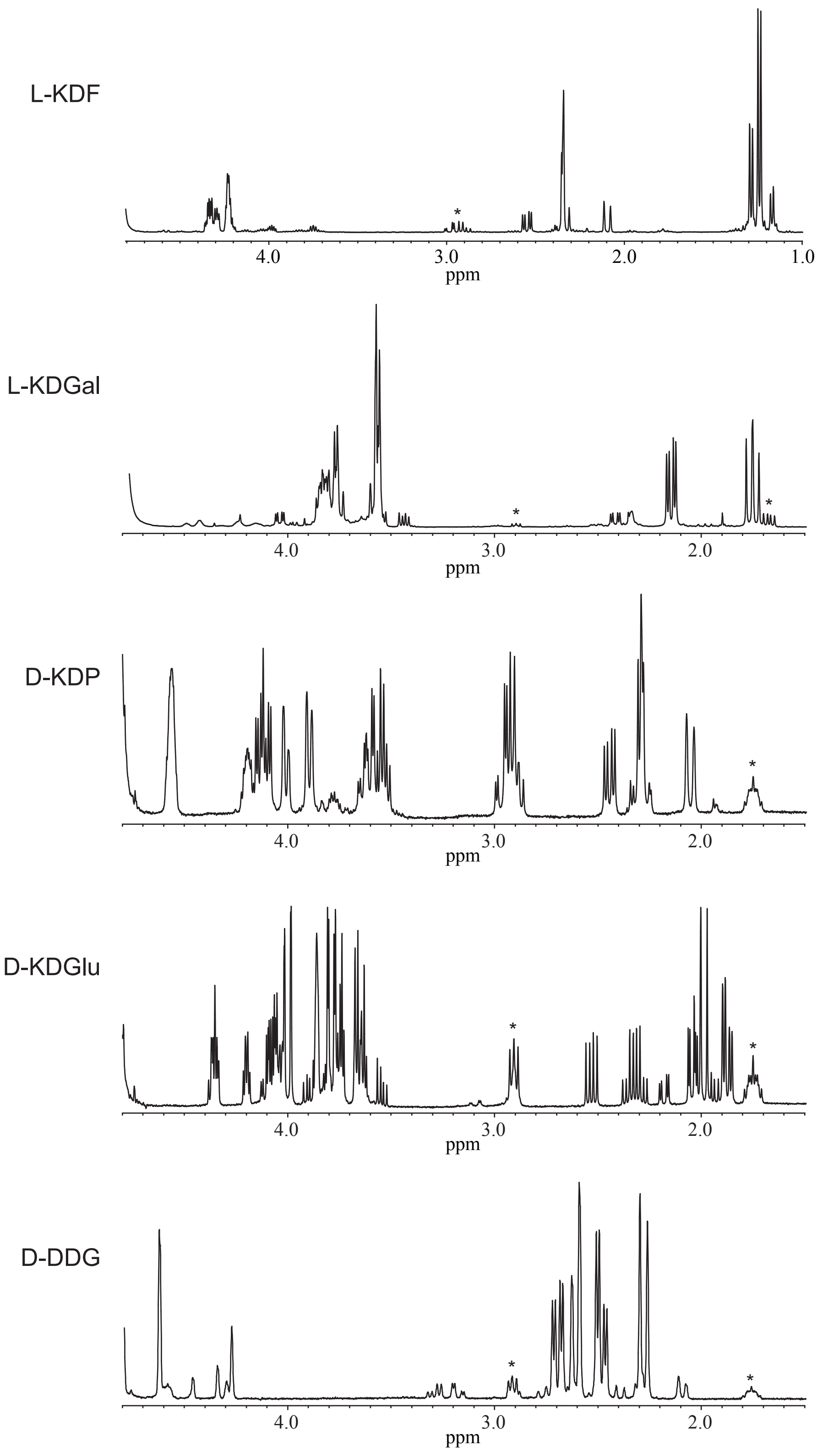

Figure S9. ^{1}H NMR spectra of 2-keto-3-deoxysugar acid substrates for L-KDFDH.
Asterisks indicate peaks derived from an internal standard.
